# Unlocking the Potential of Gallic Acid-Based Metal Phenolic Networks for Innovative Adsorbent Design

**DOI:** 10.3390/molecules30061218

**Published:** 2025-03-08

**Authors:** Shella Permatasari Santoso, Artik Elisa Angkawijaya, Kuan-Chen Cheng, Shin-Ping Lin, Hsien-Yi Hsu, Chang-Wei Hsieh, Astrid Rahmawati, Osamu Shimomura, Suryadi Ismadji

**Affiliations:** 1 Chemical Engineering Department, Faculty of Engineering, Universitas Katolik Widya Mandala Surabaya, Jl. Kalijudan 37, Surabaya 60114, East Java, Indonesia; shella@ukwms.ac.id; 2 Chemical Engineering Master Program, Widya Mandala Surabaya Catholic University, Kalijudan 37, Surabaya 60114, East Java, Indonesia; 3 Collaborative Research Center for Zero Waste and Sustainability, Jl. Kalijudan 37, Surabaya 60114, East Java, Indonesia; 4 RIKEN Center for Sustainable Resource Science, Yokohama 230-0045, Japan; artikelisa.angkawijaya@riken.jp; 5 Institute of Biotechnology, National Taiwan University, #1 Roosevelt Rd., Sec. 4, Taipei 10617, Taiwan; kccheng@ntu.edu.tw; 6 Department of Optometry, Asia University, 500, Lioufeng Rd., Wufeng, Taichung 41354, Taiwan; 7 Graduate Institute of Food Science and Technology, National Taiwan University, 1 Roosevelt Rd., Sec. 4, Taipei 10617, Taiwan; 8 Department of Medical Research, China Medical University Hospital, China Medical University, 91 Hsueh-Shih Rd., Taichung 40402, Taiwan; 9 School of Food Safety, Taipei Medical University, 250 Wu-Hsing Street, Taipei 11031, Taiwan; splin0330@tmu.edu.tw; 10 TMU Research Center for Digestive Medicine, Taipei Medical University, 250 Wu-Hsing Street, Taipei 11031, Taiwan; 11 Research Center of Biomedical Device, Taipei Medical University, 250 Wu-Hsing Street, Taipei 11031, Taiwan; 12 School of Energy and Environment, Department of Materials Science and Engineering, Centre for Functional Photonics (CFP), City University of Hong Kong, Kowloon Tong, Hong Kong, China; sam.hyhsu@cityu.edu.hk; 13 Shenzhen Research Institute of City University of Hong Kong, Shenzhen 518057, China; 14 Department of Food Science and Biotechnology, National Chung Hsing University, 145 Xingda Rd., South Dist., Taichung 40227, Taiwan; welson@nchu.edu.tw; 15 Department of Applied Chemistry, Osaka Institute of Technology, 5-16-1 Omiya, Ashahi-ku, Osaka 535-8585, Japan; veronica.astrid26@gmail.com (A.R.); osamu.shimomura@oit.ac.jp (O.S.)

**Keywords:** surface modification, adsorbent composite, chelate complex, metal phenolic coating, metal complex

## Abstract

Metal phenolic networks (MPNs) have attracted significant attention due to their environmentally benign nature, broad compatibility, and universal adhesive properties, making them highly effective for modifying adsorbent surfaces. These supramolecular complexes are formed through the coordination of metal ions with natural phenolic ligands, resulting in stable structures while retaining the active adsorption sites of the ligands, thereby enhancing the adsorption performance of unmodified substrates. Among various MPNs, metal ion gallic acid (GA) networks are particularly well-known for their exceptional stability, biological activity, and superior adsorption ability. This review offers a comprehensive examination of GA-based MPN adsorbents, focusing on their formation chemistry, characterization techniques, and applications. The coordination chemistry underlying the stability of GA–metal complexes is analyzed through equilibrium studies, which are critical for understanding the robustness of MPNs. The main analytical methods for assessing metal ligand interactions are discussed, along with additional characterization techniques for evaluating adsorbent properties. This review also explores various synthesis and performance enhancement strategies for GA-based MPN adsorbents, including stand-alone MPNs, MPN-mediated mesoporous materials, MPN-MOF composites, and MPN-coated substrates. By consolidating current advancements in MPN-based adsorbents and offering fundamental insights into their chemistry and characterization, this review serves as a valuable resource for researchers seeking to develop stable, functional metal-organic materials. It aims to drive innovation in sustainable and efficient adsorbent technologies for diverse environmental and industrial applications.

## 1. Introduction

Surface modification is a widely adopted strategy to introduce specific functional groups into adsorbents, enhancing their adsorption capacity [1,2], selectivity [3], reactivity [1,4], and stability [5]. This approach offers a practical and economical alternative to commercial activated carbon, which is often expensive. By modifying low-cost and locally available materials such as clays, zeolites, or cellulosic biomasses, adsorbents with increased active adsorption sites and improved capacity can be developed. Among the various modification techniques, surface coating stands out as a simple yet effective method. In particular, metal-phenolic network (MPN) coatings have emerged as a promising and sustainable approach [6,7,8,9,10]. In adsorption-based applications, MPN coatings introduce additional active sites through their metal and organic components [6,11], while their supramolecular structure ensures robust interconnections between the adsorbent, metal, and organic modifier. This not only enhances adsorption performance but also prevents leaching and secondary contamination, making MPN coatings a viable solution for sustainable adsorbent development.

MPNs are formed through the coordination of phenolic compounds with metal ions, creating stable metal-organic complexes. Due to their universal adherence properties, MPNs offer remarkable versatility in surface modification across various materials, including adsorbents [12,13]. Tannic acid has become the most extensively studied phenolic compound for MPN preparation, particularly following the pioneering work of Ejima et al. (2013) [14]. However, the predominant focus on tannic acid-based MPNs has overshadowed the potential of other phenolic compounds, such as gallic acid (GA), which remains underexplored despite its promising structural and functional attributes.

Gallic acid (GA), chemically known as 3,4,5-trihydroxybenzoic acid, is a naturally occurring polyphenol with a planar molecular structure. It exhibits an aromatic ring and galloyl group, which consists of one carboxylic group and three hydroxyl phenol groups. This galloyl group enables GA to function as a strong organic linker, chelating metal ions through a coordination self-assembly process [15,16,17]. This interaction leads to the formation of metal complexes with adhesive properties, capable of forming thin films on various substrate surfaces upon coordination with metal ions [18].

While previous reviews have extensively covered MPNs in general [13,19], studies specifically dedicated to GA-based MPNs remain scarce. This review provides the first comprehensive evaluation of GA-based MPNs for adsorbent preparation. The review begins by elucidating the coordination chemistry of GA-based metal complexes, offering critical insights into metal ligand interactions and the selection criteria for organic linkers. Next, the review examines the stability of GA-based metal ligand complexes, a fundamental yet often overlooked aspect of MPN chemistry that directly influences adsorbent performance. Additionally, key characterization techniques used to confirm metal-complex formation are discussed.

Furthermore, this review explores the potential of GA-based MPNs in adsorbent preparation, summarizing various MPN-based adsorbents derived from GA, including standalone MPN adsorbents, MPN-mediated mesoporous materials, MPN-MOF composites, and MPN-coated substrates. By integrating fundamental principles with practical insights, this review addresses a critical knowledge gap and offers a structured framework for researchers developing sustainable and high-performance MPN-based adsorbents. Through an in-depth discussion of the coordination chemistry and stability of GA-based MPNs, it establishes a foundation for future advancements in MPN-modified adsorption materials.

## 2. Evolution of Gallic Acid-Based Metal Complexes

**Figure 1:**
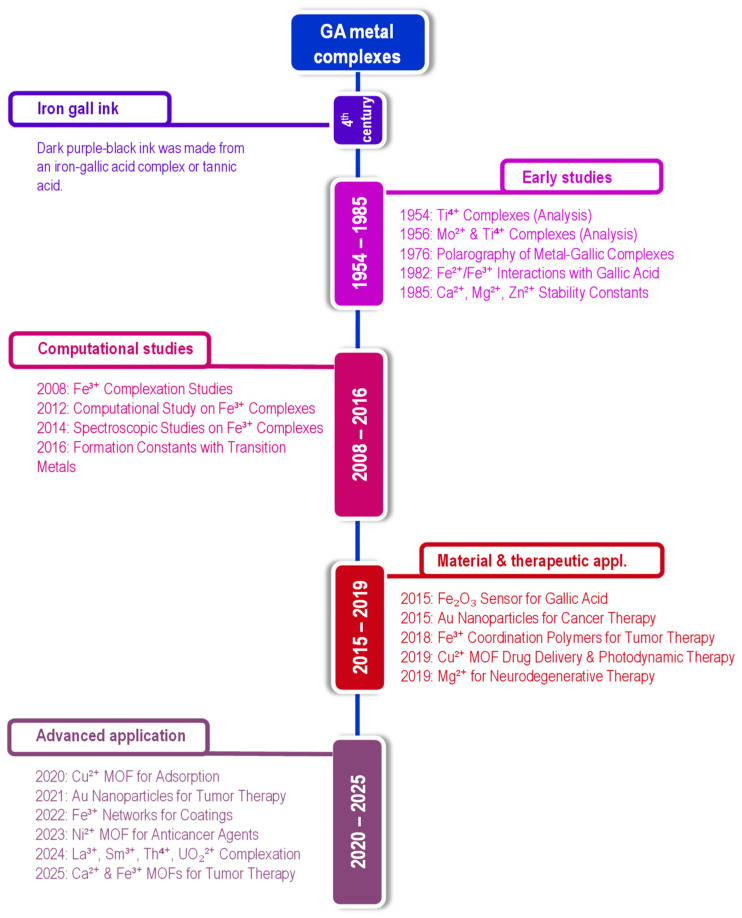
Timeline illustrating the development of gallic acid-based metal complexes, compiled from the publication list in Supplementary Information Table S1.

The study of gallic acid-based metal complexes has evolved from fundamental coordination chemistry to advanced applications in material science, therapeutics, and environmental technology. Figure 1 illustrates a timeline highlighting the evolution of gallic acid-based metal complexes and their applications in multifunctional fields.

Early research (1954–1985) focused on elucidating the coordination chemistry of gallic acid-metal interactions, particularly with Ti^4+^ [20,21], Mo^2+^ [22], Fe^2+^, Fe^3+^ [23], Ca^2+^, Mg^2+^, and Zn^2+^ [24,25]. With advancements in computational modeling and spectroscopy between 2008 and 2016, researchers refined their understanding of transition metal coordination [17], protonation equilibria [26], and electron distribution in GA-based metal complexes [27]. These studies extended beyond analytical chemistry to applications in cultural heritage preservation [28] and antioxidant research [29]. Due to its high metal-binding affinity, GA has also been explored for metal detection in solutions and soil, playing a crucial role in phytoremediation, where GA-rich plants enhance metal sequestration from contaminated environments [30].

Between 2015 and 2020, research expanded into biomedical and material science applications. GA-metal complexes were employed in electrochemical sensors [29], cancer therapy [31], and neurodegenerative treatments [32]. The emergence of metal-organic frameworks (MOFs) and metal phenolic networks (MPNs) revolutionized drug delivery and photodynamic therapy [33]. MPNs, in particular, gained attention for their biocompatibility, self-assembly properties, and redox-responsive behavior, making them highly effective in biomedical coatings and controlled drug release [34]. During this period, adsorption applications also gained prominence, particularly in environmental remediation. GA-based MOFs, such as Zr- and Cu-based frameworks [35,36,37], demonstrated excellent performance in enhanced mass spectrometry and dye removal from wastewater, leveraging GA’s strong affinity for metal ions and organic pollutants.

Recent studies have focused on MOFs for wastewater treatment, targeted drug delivery, and sustainable materials. The development of gallic acid-calcium grafts [38] and bismuth-gallic acid MOFs [39] has enhanced tumor therapy and tissue regeneration. Meanwhile, MPNs have evolved into tunable coatings, mimicking natural protective barriers and expanding their applications in biomedicine [40] and advanced materials. Adsorption technologies have continued to advance, with AI-optimized green synthesis of GA-based MOFs enhancing efficiency in heavy metal and dye removal [41]. Additionally, GA’s role in metal sequestration and phytoremediation continues to grow, reinforcing its importance in environmental sustainability.

The evolution of GA-based metal complexes reflects their versatility, from historical ink formulations and metal detection to cutting-edge biomedical, adsorption, and environmental applications. Among these developments, MPNs and MOFs stand out as transformative innovations, unlocking new possibilities in biomedicine, coatings, adsorption technologies, and sustainable materials.

## 3. Gallic Acid-Based Metal Complexes, an MPN or MOF?

MOFs and MPNs are supramolecular materials composed of metal ions or clusters coordinated with multidirectional organic ligands. Their shared precursors often lead to the misconception that they are interchangeable, yet their structural and synthetic distinctions are significant. MOFs are highly crystalline, exhibiting well-organized frameworks that provide exceptional porosity and internal surface area [42]. Their formation typically requires heat and pressure [43,44,45], though some MOFs can be synthesized under milder conditions, often resulting in varied particle morphologies [46,47]. In contrast, MPNs are amorphous coordination compounds that form spontaneously under ambient conditions [19]. They exist as molecular species in solution but can aggregate into solid structures at high precursor concentrations or prolonged reaction times. Unlike MOFs, which often adopt well-defined geometric shapes (e.g., cubic, octahedral, or tetrahedral), MPNs generally lack distinct morphological features due to their cluster-based assembly.

**Figure 2:**
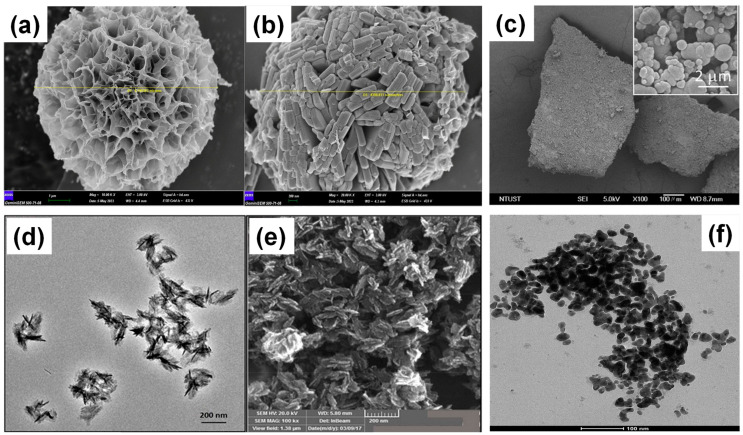
(**a,b**) Morphological characterization of gallic acid (GA)-based metal complexes. SEM images of supramolecular materials formed through coordination between GA and copper (**a**) and GA and zinc (**b**). Images reproduced from Yilmaz (2024) [48], under a Creative Commons 4.0 license. (**c**) SEM image of N-grafted GA-copper MPN. Image adapted from Santoso et al. (2021) [37], licensed under CC-BY. (**d**,**e**) TEM and SEM images of copper-gallic acid metal-organic framework. Image reprinted from Sharma et al. (2019) [33], with permission from the American Chemical Society. (**f**) TEM image of iron gallate particles. Image reproduced from Yadav et al. (2019) [49], with permission from Elsevier B.V. All rights reserved.

For GA-based metal complexes, their behavior aligns more closely with MPNs. While GA-metal systems can remain in solution, they can also form solid frameworks resembling MOFs. For instance, Yilmaz (2024) reported the formation of two metal complexes with nanoflower-like structures, synthesized by reacting GA and copper at 4 °C for 3 days, and the other by reacting GA and zinc at 25 °C for 24 h [48]. These materials exhibited broad-spectrum antimicrobial activity and anticancer properties. The solid formation of these materials likely resulted from the uninterrupted clusterization of metal-GA networks, which aggregated into supramolecular structures (Figure 2a,b). Similarly, Santoso et al. (2021) synthesized spherical MPN particles from Cu^2+^ and GA, with N-functionalization achieved through glycine addition (Figure 2c) [37]. Meanwhile, Sharma et al. (2019) produced Cu-gallate solids with nanorod morphology by reacting copper acetate and GA in DMF with a micellar surfactant at 70–80 °C for 12 h [33]. The resulting material exhibited polycrystalline XRD patterns akin to MIL-53 and aggregated rod-like morphology (Figure 2d,e).

MOFs are renowned for their exceptionally high internal surface areas, which contribute to their superior porosity and gas adsorption properties. Notable examples include Zr-MOF NU-1103 (6550 m^2^/g) [50], Cu-MOF HKUST-1 (1850 m^2^/g) [51], and Cu-tbo-MOF-5 (3971 m^2^/g) [52], all of which demonstrate excellent gas storage potential. In contrast, MOFs with lower surface areas are often employed for aqueous-phase adsorption. For instance, MIL-101(Fe)/WO_3_ (20.74 m^2^/g) effectively adsorbs tetracycline-HCl from solution [53], MIL-101(Fe)/Bi_2_MoO_6_ (39.72 m^2^/g) exhibits both photodegradation and photofixation capabilities toward tetracycline-HCl [54]. For GA-based metal complexes, surface area variations significantly influence adsorption performance. A nitrogen-grafted CuGA complex, despite its low surface area (2.00 m^2^/g) [37]. In contrast, a non-grafted CuGA complex, with a higher surface area (198.22 m^2^/g), shows slightly lower adsorption efficiency for the same dye [36]. NiGA exhibits a surface area of 196 m^2^/g, showing antimicrobial and anticancer activity [55].

These studies highlight the nuanced classification of GA-based metal complexes. While they often align with MPNs due to their amorphous nature, mild synthesis conditions, and spontaneous self-assembly, some may resemble MOFs. Additionally, GA’s susceptibility to oxidation under high-temperature conditions limits its suitability as a ligand for MOF synthesis. Collectively, these factors underscore the advantages of MPNs, particularly for applications that demand flexible and low-energy synthesis.

## 4. Coordination Chemistry of GA-Based Metal Complexes

### 4.1. Protonation and Deprotonation of Ligands

**Figure 3:**
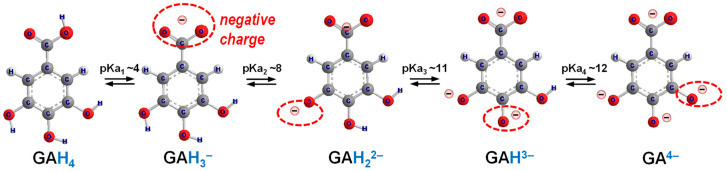
Stepwise ionization constants of gallic acid (GA). GAH_4_ represents the fully protonated molecule. Each deprotonation step leads to the sequential formation of negatively charged species: GAH_3_^−^, GAH_2_^2−^, GAH^3−^, and GA^4−^. In the molecular structure, oxygen atoms are represented by red spheres, hydrogen atoms by white spheres (with larger spheres indicating non-ionizable hydrogen atoms and smaller spheres indicating ionizable hydrogen atoms), and carbon atoms by grey spheres. The red dashed circles highlight the sites of subsequent deprotonation.

Understanding the ionization behavior of ligands is critical for elucidating the formation of chelate complexes [56]. Phenolic compounds, as ligands, possess ionizable functional groups that can undergo protonation or deprotonation, processes that are strongly modulated by pH. In the case of gallic acid (GA), it contains four ionizable groups, that is, one carboxylic moiety and three hydroxy phenolic moieties [57,58]. The carboxylic moiety, being the most acidic group, deprotonates first as the pH increases, followed by the sequential deprotonation of the hydrogen ions (H^+^) from the three hydroxy phenolic moieties. This stepwise deprotonation of GA can be expressed by the following equilibrium Equations (1)–(4) and is illustrated in Figure 3:

(1) $${GAH}_{4}⇌H^{+}+{GAH}_{3}^{-}\;{[H}^{+}]\left[{GAH}_{3}^{-}\right]={Ka}_{1}[{GAH}_{4}]$$

(2) $${GAH}_{3}^{-}⇌H^{+}+{GAH}_{2}^{2-}\;{[H}^{+}]\left[{GAH}_{2}^{2-}\right]={Ka}_{2}[{GAH}_{3}^{-}]$$

(3) $${GAH}_{2}^{2-}⇌H^{+}+{GAH}^{3-}\;{[H}^{+}]\left[{GAH}^{3-}\right]={Ka}_{3}[{GAH}_{2}^{2-}]$$

(4) $${GAH}^{3-}⇌H^{+}+{GA}^{4-}\;{[H}^{+}]\left[{GA}^{4-}\right]={Ka}_{4}[{GAH}^{3-}]$$

The protonated ligand is designated by the general formula AH*_x_^z^*, where A denotes the ligand abbreviation (e.g., GA for gallic acid), H represents the proton, *x* is the number of protons associated with all ionizable groups, and *z* is the net charge of the species at its current state. The protonation constant (Ka, also referred to as acidity constant, ionization constant, or acid dissociation constant) reflects the relative capacity of a compound to donate a proton [59]. Its logarithmic value, pKa, indicates the pH at which protonation or deprotonation of the ionizable group occurs.

In its fully protonated state, GA possesses four protons, one at each ionizable group, and is denoted as GAH_4_, indicating four protons and a neutral charge. The first deprotonation occurs at the carboxylic group, with a pKa_1_ value approximately 4, forming the negatively charged species of GAH_3_^−^. The second deprotonation occurs at one of the hydroxy groups, with a pKa_2_ value of approximately 8, forming GAH_2_^2−^. The third and fourth deprotonations, with pKa_3_ and pKa_4_ values of approximately 11 (GAH^3−^) and 12 (GA^4−^), respectively, require highly alkaline conditions.

Table 1 summarizes the reported pKa values of GA from various publications, ranging from older to more recent studies. The reported values are generally consistent for pKa_1_ and pKa_2_. However, significant variations in the reported pKa_3_ and pKa_4_ values is likely due to the extreme alkaline conditions required for accurate determination. Additionally, discrepancies may arise from differences in experimental conditions, such as the use of different salts and ionic strengths. For instance, salts such as KCl are highly reactive and can significantly influence ionization behavior.

### 4.2. Metal Complex Formation and Stability Constant

The interaction between ligands and metal ions is governed by the specific functional groups of the ligand, as well as the charge density and polarizability of the metal ion. According to Pearson’s Hard and Soft Acids and Bases (HSAB) theory (see Supplementary Information Table S2), metal ions act as Lewis acids (electron acceptors), while ligands function as Lewis bases (electron donors) [63]. Hard acids, characterized by high charge density and low polarizability, preferentially bind to hard bases through ionic interactions. In contrast, soft acids, which have low charge density and high polarizability, form covalent bonds with soft bases. Gallic acid (GA), with its hydroxyl (–OH) functional groups, behaves as a hard base, forming stable coordination bonds with hard acids such as Fe^3+^ [17,27], a metal ion commonly used in metal-phenolic network (MPN) synthesis. While GA can also coordinate with borderline and soft acids, these complexes exhibit lower stability.

Crystal field theory (CFT) further explains metal ligand stability through ionic potential (charge-to-radius ratio), underpinning the Irving–Williams Series (IWS). IWS predicts the stability trend of first-row transition metal complexes (i.e., Mn^2+^ to Zn^2+^) [64]. According to IWS, decreasing ionic radius from Mn^2+^ to Zn^2+^ leads to increased stabilization energy, enhancing complex stability. However, Cu^2+^ deviates from this trend due to the Jahn-Teller effect [65], which provides additional stabilization, making Cu^2+^ complexes more stable than those of Ni^2+^. The general stability trend follows: Mn^2+^ < Fe^2+^ < Co^2+^ < Ni^2+^ < Cu^2+^ > Zn^2+^.

To quantitatively assess metal ligand interaction strength, the stability constant (log*K*) is a key parameter for predicting complex formation and stability [66,67], serving as a fundamental metric for evaluating MPN stability. The stepwise stability constant (*K_n_*) for each stage of metal ligand complexation is expressed as Equation (5).

(5) $$ML_{n-1}+L⇌ML_{n}\;K_{n}=\frac{\left[ML_{n}\right]}{\left[ML_{n-1}\right]\left[L\right]}$$

where M is the metal ion, L is the ligand, and *n* represents the number of ligands coordinated to the metal. The overall stability of the metal complex is represented by the cumulative stability constant (log*β*), given by Equation (6).

(6) $$M+nL⇌ML_{n}\;\beta_{n}=\frac{\left[ML_{n}\right]}{\left[M\right]{\left[L\right]}^{n}}$$

$$\beta_{n}=K_{1}\times K_{2}\times K_{3}\times \ldots \times K_{n}=\sum_{i=1}^{n}K_{i}$$

where *β_n_* denotes the overall formation constant for a complex with n ligands. The higher the *β_n_* value, the greater the stability of the resulting metal ligand complex, making it a crucial factor in determining the feasibility of MPN formation and adsorption efficiency.

Before complex formation, ligand deprotonation occurs, enhancing its electronegativity and facilitating metal ion attraction [68]. The metal-to-ligand ratio and pH are key factors affecting complex type and stability. Table 2 presents the log*K* values for complex formation between GA and various transition metal ions, such as Cu^2+^, Zn^2+^, Ni^2+^, Fe^3+^, and Co^2+^. Among these ions, Fe^3+^, with its higher charge density and smaller ionic radius, facilitates strong binding with GA, leading to the formation of the most stable complex, aligning with HSAB theory. The observed GA-metal stability trend (Co^2+^ < Ni^2+^ < Cu^2+^ > Zn^2+^) also aligns with the IWS prediction.

Phenolic compounds, including GA, can form complexes with metal ions, but the stability of these complexes varies significantly and must be carefully evaluated. Not all phenolic compounds form highly stable complexes. For instance, Radalla (2015) reported log*K* values for several phenolic acids complexed with transition metal ions, demonstrating that GA complexes exhibit the highest stability among phenolic acid ligands [61]. Similarly, Sursyakova et al. (2017) demonstrated that GA forms more stable complexes than succinic acid, with log*K* values of 2.89 for the CuGA complex and 3.88 for Cu(GA)_2_, with CuGA being the predominant species in the pH range of 3.5 to 8 [69]. The stability of metal ligand complexes is heavily influenced by the nature of the ligand. Some ligands form only sigma (σ) bonds with metal ions, whereas others, such as GA, can form both σ and pi (π) bonds. Ligands containing a benzene ring are particularly effective due to their ability to donate electrons through π-symmetric interactions, which not only influence the geometrical configuration but also significantly enhance the stability of the resulting complexes [70,71].

## 5. Analysis Techniques for Predicting the Metal Complex Formation

MPNs are (supra)molecular structures that form in aqueous solutions through the coordination of metal ions with organic ligands. The process begins with the deprotonation of the ligand, generating a negatively charged ligand field that strongly attracts positively charged metal ions. This deprotonation, involving the release of protons from the ligand, can be quantitatively analyzed using potentiometric titration, where each ionizable group’s deprotonation appears as an inflection point in the titration curve. The presence of metal ions often facilitates proton release, shifting the titration curve toward lower pH values and providing valuable insights into the complexation process.

To characterize metal ligand complex formation at the molecular level, advanced analytical techniques are crucial. Spectroscopic methods such as Raman, electron paramagnetic resonance (EPR), UV-Vis, Fourier-transform infrared (FTIR), and nuclear magnetic resonance (NMR) spectroscopy are particularly effective in evaluating complex formation. These techniques offer detailed structural and electronic information, enabling a comprehensive understanding of the coordination chemistry governing MPN assembly.

### 5.1. Potentiometric Titration

**Figure 4:**
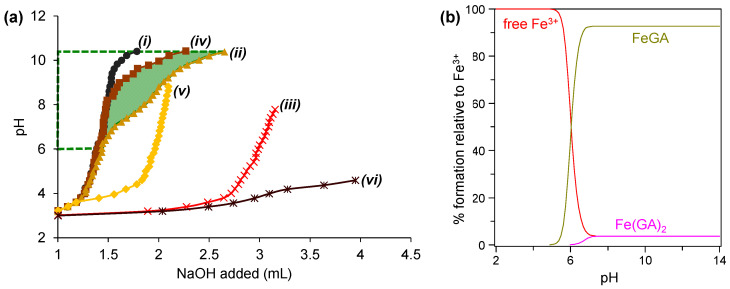
(**a**) Titration curves of a solution containing gallic acid, Fe^3+^, and glycine at 0.1 M ionic strength: (*i*) 3 mM HNO_3_, (*ii*) solution *i* + 1 mM gallic acid (ligand only), (*iii*) solution *ii* + 0.4 mM Fe^3+^, (*iv*) solution *i* + 1 mM glycine, (*v*) solution *iv* + 0.4 mM Fe^3+^, (*vi*) solution *i* + 1 mM gallic acid + 1 mM glycine + 0.4 mM Fe^3+^. The green shaded area highlights the shift in the gallic acid curve resulting from the addition of Fe^3+^. The figure was redrawn from Ref. [17] by extracting data points using Origin 2024(10.1) software. The data points for drawing these curves are provided in Supplementary Table S3. (**b**) Species distribution curve as a function of pH for GA-Fe^3+^ system, with percentages of formation relative to Fe^3+^. The figure was created using HySS2009 software to model metal ligand complex distribution, with input pKa and logK values obtained from Ref. [17]. The data points for drawing the curve are provided in Supplementary Table S4.

Potentiometric titration is a robust and widely used analytical technique for predicting metal ligand complex formation across a broad pH range. This method provides critical insights into the likelihood of complexation by analyzing changes in protonation behavior and stability constants. Figure 4a illustrates the potentiometric titration curves for the titration of Fe^3+^ with GA (and/or glycine ligand). The protonation constant (pKa) of GA and the stability constant (log*K*) of its metal complex can be determined by analyzing the difference in the volume of NaOH required to achieve the same pH value in titration curves (*i*) and (*ii*) for pKa, and (*i*) and (*iv*) for log*K*. Similarly, for glycine, pKa and logK are derived from the differences in NaOH consumption between curves (*i*) and (*iii*) and curves (i) and (v), respectively, following the Irving and Rossotti methodology [17]. The formation of metal complexes is evidenced by the shift in the titration curve for the system containing Fe^3+^ and GA (curve *iv*) to lower pH values compared to the curve for GA only (curve *i*), as highlighted by the green shaded area in Figure 4a. This shift indicates the binding of Fe^3+^ to GA, which alters the protonation behavior of the ligand.

**Figure 5:**
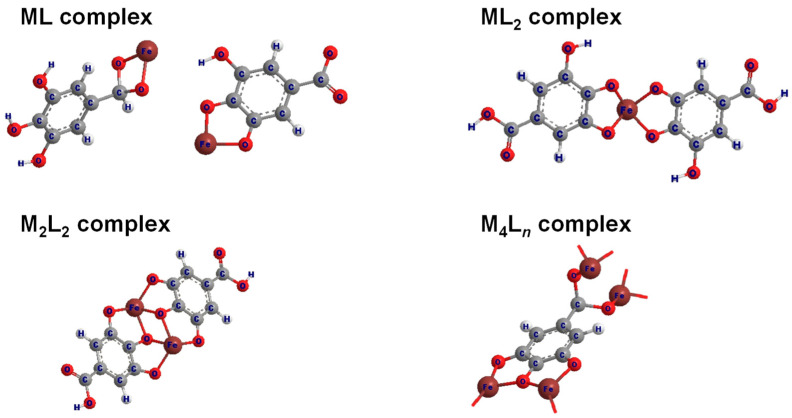
Structural representation of typical binary Fe^3+^-GA complexes. Typical binary complexes are formed with metal-to-ligand ratios of 1:1 (FeGA), 1:2 (Fe(GA)_2_), and ratios such as Fe_2_(GA)_2_ and Fe_4_(GA)*_n_*. Structural illustrations adapted and redrawn from Refs. [17,75]. In the molecular structure, oxygen atoms are represented by red spheres, hydrogen atoms by white spheres (with larger spheres indicating non-ionizable hydrogen atoms and smaller spheres indicating ionizable hydrogen atoms), carbon atoms by grey spheres, and iron atoms by the brown spheres.

Using the open-access Hyperquad Simulation and Speciation 2009 (HySS2009) software [72], the log*K* values corresponding to the stability constants of the metal complexes can be stimulated, allowing for visualization of the distribution of metal complex species across various pH ranges [73]. Illustrated in Figure 4b is the species distribution of Fe^3+^ and GA complexes. FeGA begins forming at pH 5.0 and is predominantly abundant between pH 7.0 and highly alkaline pH. This observation aligns well with the shift observed in the titration curve in Figure 4a. In contrast, Fe(GA)_2_ forms at lower abundance in the same pH range, due to its reduced stability (lower log*K*), which is attributed to weaker electrostatic interactions between Fe^3+^ and the second ligand molecules [74]. The structural representation of the typical Fe^3+^-GA complexes at various metal-to-ligand ratios is presented in Figure 5.

The formation of the complexes is influenced by two primary factors: (i) pH, which dictates ligand deprotonation and the availability of deprotonated groups for coordination, and (ii) metal-to-ligand ratios, where higher metal or ligand concentrations can favor the formation of complexes with higher stoichiometry. At acidic pH (2.0–6.0), uncoordinated Fe^3+^ and GA, dominate because the ligand remains mostly protonated, limiting strong electrostatic interactions. At pH > 6.0, abundant deprotonated ligand species readily coordinate with Fe^3+^, explaining why MPN synthesis typically occurs under alkaline conditions. However, while FeGA persists under extreme alkaline pH, synthesis in such conditions is impractical due to the need for excessive alkaline compounds. High OH^−^ concentrations can lead to the formation or precipitation of metal hydroxide, especially in concentrated metal ligand solutions often used in MPN synthesis. This highlights the importance of optimizing synthesis conditions to promote effective complex formation.

### 5.2. Spectrophotometric-Based Analyses

Spectrophotometric techniques are well suited to analyze metal complexes because of their unique electronic structures and optical properties, which arise from the interactions between metal ions and ligands. Crystal field theory (CFT) and ligand field theory (LFT) provide the basic framework for understanding these properties, explaining how ligands create electric fields that partition d orbitals into different energy levels and how molecular orbital interactions influence electronic transitions [76,77]. Transition metal complexes, especially those involving d- and f-block elements, exhibit diverse oxidation states and electronic configurations, leading to characteristic transitions such as d-d, charge transfer, and ligand-centered transitions, all of which can be examined using spectrophotometry. Advanced computational methods, including molecular orbital (MO) and density functional theory (DFT), further enhance the ability to predict and interpret absorption and emission spectra, offering insights into stability, reactivity, and bonding interactions [76,78]. These capabilities make spectrophotometry a versatile and indispensable tool, playing a crucial role in advancing research in areas such as analytical chemistry, supramolecular chemistry, and the development of functional materials such as metal-organic frameworks (MOFs), molecular machines, including MPNs [76,77,79,80].

#### 5.2.1. Raman Spectroscopy

**Figure 6:**
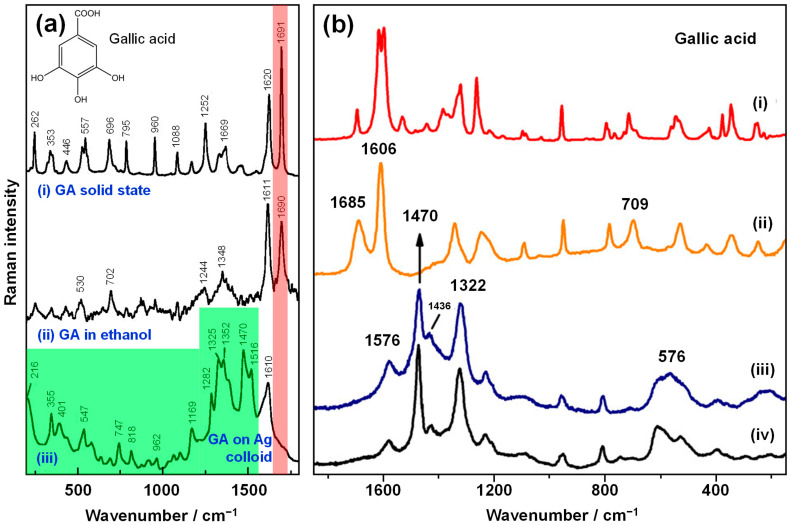
(**a**) Raman spectra illustrating the interaction of gallic acid (GA) with silver (Ag) in a colloidal system: (i) solid GA, (ii) 60 mM GA in ethanol, and (iii) 5 mM GA on Ag colloid at pH 3.9. The red-highlighted region corresponds to the vibrational modes of the carboxylic group in GA, which disappear due to deprotonation upon dissolution in ethanol. The green-highlighted region represents new spectral bands in GA induced by its interaction with the Ag colloid. Image adapted from Sánchez-Cortés and García-Ramos (2000) [82], with permission from Academic Press. All rights reserved. (**b**) Raman spectra of the metal complex between gallic acid (GA) and Fe^3+^: (i) solid GA, (ii) 20 mM GA in aqueous solution, (iii) Fe-GA complex on paper, and (iv) Fe-GA complex in solution at a ligand-to-metal molar ratio of 1:3 and pH of 7. Image adapted from Espina et al. (2022) [83], licensed under CC-BY-NC-ND 4.0.

Raman spectroscopy, particularly when coupled with Surface-Enhanced Raman Spectroscopy (SERS), serves as a highly sensitive and powerful tool for probing the functional groups of ligands involved in interactions with metal ions during the formation of metal complexes. These vibrational spectroscopy techniques enable the detection and identification of target analytes at the single-molecule level, offering unparalleled insights into molecular interactions [81]. For instance, Sánchez-Cortés and García-Ramos (2000) demonstrated the effectiveness of these techniques by examining the spectral changes in GA upon its interaction with Ag colloid [82]. In their study, the band corresponding to the carboxylic (–COOH) group, observed at 1690 cm^−1^ in the solid-state spectrum of GA (Figure 6(ai)), disappeared in the SERS spectrum of the GA dissolved in ethanol (Figure 6(aii), red-highlighted spectrum). This disappearance indicates the ionization of the –COOH group at neutral pH. Additionally, the SERS spectrum of GA on Ag colloid (Figure 6(aiii), green-highlighted spectrum) revealed the emergence of new spectral features, reflecting chemical changes in GA induced by the presence of the Ag colloid. These spectral alterations are consistent with the formation of metal ligand coordination complexes, highlighting the utility of SERS in elucidating such interactions.

Similarly, Espina et al. (2022) investigated the Raman spectra of iron gall ink species, specifically the Fe-GA complex, and observed significant restructuring of the vibrational spectra between free GA and metal-complexed GA (Figure 6(bi,bii) compared to Figure 6(biii,biv)) [83]. Three prominent bands at 1470 cm^−1^, 1322 cm^−1^, and 576 cm^−1^ were identified as characteristic of iron gall ink species, with these bands being widely documented in prior studies [84,85,86]. Density functional theory (DFT) calculations on the Fe-GA complex revealed that the band at 1470 cm^−1^ arises from benzene ring vibrations coupled to C–O stretching and C–H bending. This band intensifies in the spectrum of metal-complexed GA due to changes in polarizability induced by metal coordination. The band at 1322 cm^−1^ corresponds to ring stretching coupled to C–O stretching and C–H bending, while the band at 576 cm^−1^, exclusive to the metal-complexed GA, is attributed to Fe–O stretching, confirming metal coordination.

These findings underscore the exceptional capability of SERS to provide detailed insights into molecular interactions and structural transformations at the nanoscale. By capturing subtle spectral changes and correlating them with specific molecular vibrations, SERS enables a deeper understanding of the mechanisms underlying metal ligand complexation and related chemical processes.

#### 5.2.2. Fourier Transform Infrared (FTIR) Spectroscopy

**Figure 7:**
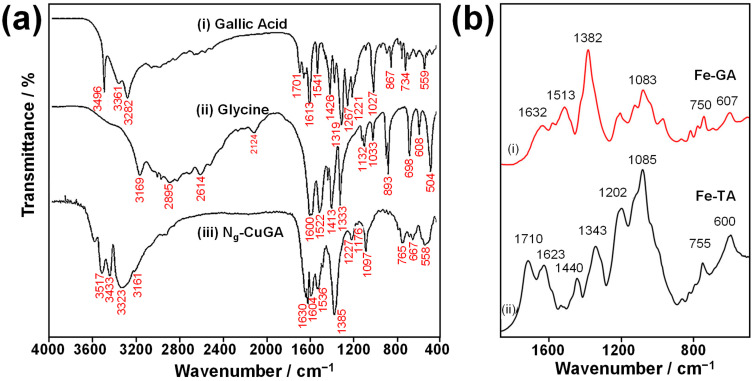
(**a**) FTIR spectra of the solid-state metal complexes formed by the coordination of Cu^2+^ with gallic acid (GA): (i) solid GA, (ii) solid glycine, and (iii) solid ternary metal complex. Image adapted from Santoso et al. (2021) [37], licensed under CC-BY. (**b**) FTIR spectra of solid-phase metal complexes of Fe^3+^ with gallic acid (GA) or tannic acid (TA): (i) Fe-GA complex and (ii) Fe-TA complex. Image adapted from Espina et al. (2022) [83], licensed under CC-BY-NC-ND 4.0.

FTIR spectroscopy is a valuable tool for confirming the successful formation of metal ligand complexes by comparing the spectra of the complex with those of the original ligand. It is particularly effective in detecting prominent vibrations of oxygen-containing functional groups. For example, Santoso et al. (2021) demonstrated the use of FTIR to confirm the formation of the metal complex N_g_-CuGA from GA and Cu^2+^, with additional nitrogen group functionalization via ternary complexation with glycine [37]. The complexation of GA with Cu^2+^ induces vibrational band shifts in the galloyl group, specifically in the –OH groups, as indicated by the shifting of the bands between the wavenumbers 3282 and 3496 cm^−1^ (Figure 7(aiii)). Furthermore, nitrogen functionalization is confirmed by the presence of a band at 3161 cm^−1^ in the N_g_-CuGA spectra, indicating the –NH group. The observed shift in this amine group’s spectral position, compared to glycine alone, suggests its involvement in metal ion coordination during complex formation.

Similarly, Espina et al. (2022) highlighted the robustness of FTIR in identifying functional groups involved in metal chelation [78]. Upon complexation with Fe^3+^, the band corresponding to C–O stretching (1309 cm^−1^) and C–OH bending (1014 cm^−1^) decreases in intensity compared to GA alone, while a strong band at 1083 cm^−1^ appears instead (Figure 7(bi)), indicating Fe^3+^ coordination with the –OH of the phenolic group. Additionally, the appearance of a strong band at 1382 cm^−1^ has been attributed to the interaction between Fe^3+^ and the –COOH group. The Fe–O coordination is observed at 607 cm^−1^ for the FeGA complex and at 600 cm^−1^ for the FeTA complex (Figure 7(bii)).

Several other works also reported changes in the spectra as induced by the complexation. Lunardi et al. (2023) reported a reduction from a doublet to a single peak near ~2900 cm^−1^ [6], while Liu et al. (2021) observed the disappearance of the O–H vibration peak at ~3300 cm^−1^ [87]. Espina et al. (2022) documented alterations in peaks within the 1300–1000 cm^−1^ range [83]. These spectral changes provide critical insights into the coordination environment and structural transformations of the ligand upon complexation.

#### 5.2.3. Electron Paramagnetic Resonance (EPR) Spectroscopy

EPR spectroscopy is a powerful technique for characterizing ligand fields (the negative point charges of ligands) and determining the oxidation states of metal complexes. It provides valuable insights into the reaction chemistry between metals and ligands across a wide range of experimental conditions [88]. For instance, Pirker et al. (2012) successfully employed EPR spectroscopy to investigate the formation of mononuclear Cu^2+^ complexes with GA [89]. Their study identified three distinct Cu-GA complex species through S-band and X-band EPR spectra, denoted as Complexes I, II, and III.

**Figure 8:**
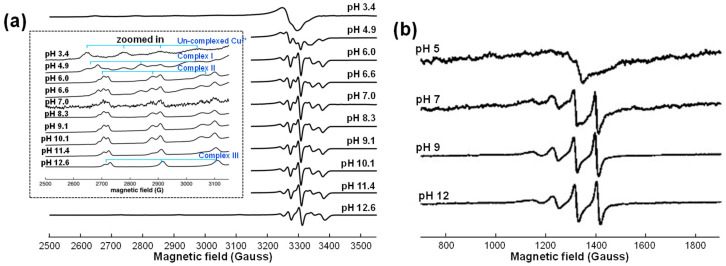
EPR spectra of gallic acid (GA) complexed with Cu^2+^ metal ions at metal-to-ligand molar ratio of 1:5 at different pH values: (**a**) S-band spectra and (**b**) X-band spectra. Images were reproduced from Pirker et al. (2021) [89], with permission from Elsevier Inc.

Complexes I and II were assigned as mono- and bis-Cu^2+^ GA complexes, respectively, with Cu^2+^ coordinating to two hydroxy-phenolic groups of GA, as illustrated in Figure 5 for ML and ML_2_ complexes. Complex III, on the other hand, was hypothesized to form through additional coordination of glycerol. The S-band EPR spectra at an acidic pH of 5 revealed the presence of hydrated, uncomplexed Cu^2+^, [Cu(H_2_O)6]^2+^. While Complex I was hardly detectable in the S-band spectra (Figure 8a), it was clearly observed in the X-band spectra (Figure 8b). At pH 7, the S-band spectra exhibited overlapping signals from Complexes II and III, whereas only Complex III was detected at higher pH values.

#### 5.2.4. UV-Vis Spectroscopy

The stability of metal ligand complexes in aqueous solutions is commonly quantified using the stability constant (log*K*), while their structural characteristics are elucidated through spectroscopic techniques. These methods are crucial for confirming the successful formation of metal phenolic networks (MPNs) or modifications induced by MPNs on substrates. The complexation of metal ions with polyphenols under varying pH conditions often results in distinct visible color changes, which arise from different modes of metal ligand coordination and the formation of diverse complex species. UV-visible absorption spectroscopy provides a straightforward means to analyze ligand-to-metal charge transfer (LMCT), which is responsible for these color changes [27,83,90,91,92].

**Figure 9:**
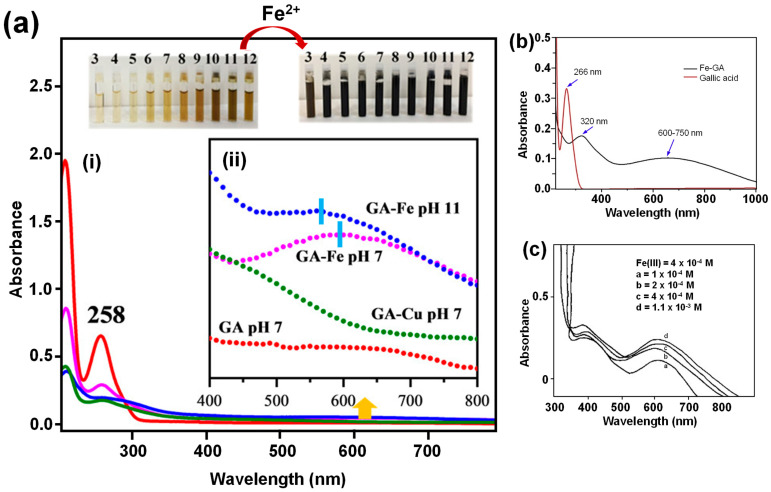
(**a**) UV-Vis absorption spectra of the complex between Fe^3+^ and gallic acid (GA): (i) 0.066 mM GA solution and (ii) metal complex at a metal-to-ligand molar ratio of 3:1. The red line represents the GA solution at pH 7, the green line represents the GA-Cu^2+^ complex at pH 7, the pink line represents the GA-Fe^3+^ complex at pH 7, and the blue line represents the GA-Fe^3+^ complex at pH 11. Image reproduced from Espina et al. (2022) [83], licensed under CC-BY-NC-ND 4.0. (**b**) Electronic absorption spectra of GA and its corresponding iron gallate nanocomplex. Image reproduced from Yadav et al. (2019) [49], with permission from Elsevier B.V. All rights reserved. (**c**) UV-Vis spectra of GA complexes with Fe^3+^ at various molar concentrations. The image was reproduced from Masoud et al. (2014) [27], with permission from Elsevier B.V. All rights reserved.

Free GA molecules exhibit two characteristic absorption bands at ~210 nm and ~260 nm, corresponding to the ^1^*L_a_* and ^1^*L_b_* singlet excited states, respectively, associated with $\pi \to \pi^{*}$ transitions. Upon complexation with metal ions at pH 7, these bands significantly decrease in intensity and may undergo a redshift. Additionally, a new, broad absorption band emerges in the visible region at ~600 nm (Figure 9(aii), indicated by the sky-blue colored bar), attributed to LMCT between the metal and the ligand [83]. This band is primarily responsible for the pronounced color changes observed during complexation. For metal complexes formed at pH 11, the band at ~600 nm undergoes a blueshift, which is attributed to the auto-oxidation of GA to quinone structures. Similar observations have been reported by Yadav et al. [49] and Masoud et al. (2014) [27], Figure 9b,c, where complexation with Fe^3+^ results in the appearance of a band in the visible range with λ_max_~600 nm.

UV-Vis spectrophotometry is a simple yet effective technique for confirming the successful coordination of metals and ligands in the formation of MPNs. This technique is applicable to various types of ligands. For instance, Mazaheri et al. (2022) [93] demonstrated the formation of Fe^3+^-tannic acid (TA) MPN through the absorption band within the range 450–650 nm with λ_max_ at 475 nm, corresponding to the formation of the mono Fe^3+^-TA complexes. This study also highlighted the stability of MPNs over prolonged aging times, noting a decrease in absorption intensity with increasing aging time due to complexation with acetonitrile, which was used as the solvent in their work.

#### 5.2.5. Nuclear Magnetic Resonance (NMR) Spectroscopy

Nuclear magnetic resonance (NMR) spectroscopy is a powerful, non-destructive analytical technique widely employed for molecular structure identification. It operates by exploiting the magnetic properties of atomic nuclei, providing detailed insights into molecular environments and interactions. In the context of metal complex characterization, NMR is particularly valuable for discerning oxidation states, coordination environments, and ligand-metal interactions. To elucidate comprehensive structural information, NMR is often combined with complementary techniques such as polarization transfer, relaxometry, and multidimensional NMR spectroscopy [80,94].

Fazary et al. (2009) have demonstrated the use of ^1^H and ^13^C NMR spectroscopy to characterize the isolated solid FeGA complex. Their analysis revealed the presence of functional ionizable groups in the ligand; however, the data did not provide conclusive evidence for the complexation between the ligand and the metal ion. In a more recent study, Etou et al. (2024) demonstrated the efficacy of liquid-state NMR spectroscopy, coupled with density functional theory (DFT) calculations, for elucidating the structure of metal complexes involving gallic acid (GA) and Al^3+^ [95]. By employing ^27^Al NMR, they compared the spectra of a solution containing only Al-salt with that of an Al^3+^-GA mixed solution. While a single peak at 0 ppm was observed for the Al-salt alone, two distinct peaks at 1.17 ppm and 15.7 ppm were detected in the Al^3+^-GA system. These peaks were attributed to the formation of monodentate and bidentate AlGA complexes, respectively, with the former arising from coordination between Al^3+^ and the –COOH group of GA, and the latter resulting from coordination with two –OH groups of GA. Further confirmation was provided by ^1^H NMR analysis, which compared the spectra of GA alone with the Al^3+^-GA mixed solution. A peak at 7.14 ppm, corresponding to GA alone, was accompanied by two additional peaks at 6.95 ppm and 7.04 ppm in the Al^3+^-GA system, consistent with the formation of the bidentate complex. This study highlights the utility of NMR spectroscopy, particularly when integrated with computational methods, for unraveling the structural intricacies of metal ligand interactions.

## 6. Versatility of GA-Based MPNs as Adsorbents

MPNs stand out as unique materials characterized by their amorphous structure, achieved through low-energy synthesis. Unlike other frameworks, MPNs are synthesized at room temperature, within short durations, and under moderate concentrations of metals and ligands. This mild synthesis process preserves the intrinsic properties of (poly)phenols, enabling strong interactions with various macromolecular substrates and metal ions. These interactions grant MPNs exceptional adhesive properties, making them highly versatile for surface engineering and adsorbent applications [96].

### 6.1. MPN as Standalone Adsorbent

**Figure 10:**
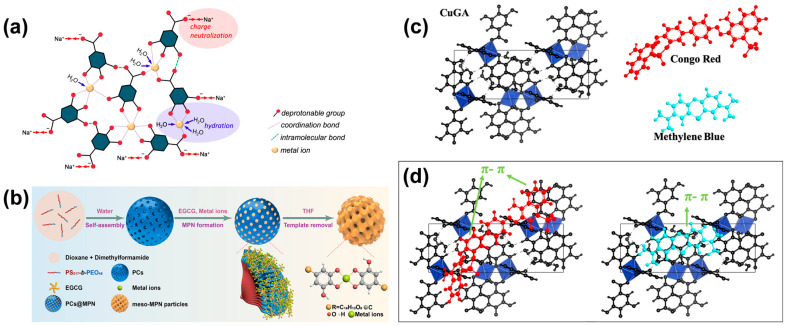
(**a**) Schematic representation of the molecular assembly process of metal phenolic networks as a standalone material. (**b**) Schematic representation of the preparation process of ordered mesoporous MPN. Adapted with permission from Lin et al. (2019) [97], Copyright © 2019, American Chemical Society. (**c**,**d**) Schematic illustration of the molecular structure of Cu^2+^-gallic acid (CuGA) complexes and their subsequent interaction mode toward Congo red and methylene blue adsorbates. Image reproduced from Azhar et al. (2020) [36], licensed under CC-BY.

MPNs have been extensively utilized as surface modifiers due to their strong adhesive properties. However, this focus has often overshadowed the exploration of MPNs as standalone materials. MPNs can be isolated as solid particles by carefully controlling parameters in the reaction, including the use of minimal amounts of solvent based on the solubility of the ligand, controlling the pH, and regulating the concentrations of metal ions and ligands during synthesis. This well-established method has been employed for decades. For instance, Patel et al. (1971) successfully isolated Ni^2+^ complexes with ethylenediamine (en) ligands, such as catechol and pyrogallol, in 1:2 and 1:3 ratios, forming Ni(en)_2_ and Ni(en)_3_. These complexes precipitated as the solution pH reached 7 upon ligand addition [60]. Ni(en)_2_ required less water and ligand, while Ni(en)_3_ necessitated larger quantities of both. Heating is sometimes applied to accelerate the formation of solid precipitates, though the temperatures required are significantly lower than those used in the synthesis of MOFs. Figure 10 illustrates the assembly of the metal complexes into MPN supramolecule. The formation of solid metal complexes resembles the agglomeration of coordinated metal ligand molecular compounds, which combine to form large clusters of supramolecular complexes. This clustering is driven by charge neutralization, which occurs as metal ligand interactions are prolonged or heating is applied. The neutralization process may also be influenced by attached water molecules to the metal ions.

A major challenge in this process is the potential decomposition of the ligand during cluster formation, particularly due to oxygen exposure. Ligand decomposition can lead to the formation of metal oxides or hydroxides instead of the desired metal ligand complex. Morales et al. (2023) reported such phenomena, noting that ligand decomposition is more likely to occur when metal acetate salts are used as the metal source [98]. The acetate ions can induce the oxidation of gallic acid (GA), leading to its decomposition into phenols, quinones, and hydroxyquinones. To prevent ligand decomposition, metal chloride salts can be used instead of metal acetates, as chloride ions do not promote oxidation.

Santoso et al. (2021) demonstrated a technique for isolating MPNs as solid particles to prepare an adsorbent [37]. The MPN adsorbent was synthesized by reacting an equimolar concentration of GA with CuCl_2_·5H_2_O in a minimal amount of water. The solid MPN precipitate was isolated by adjusting the reaction pH to 8 with NaOH. To enhance the adsorption performance, glycine was introduced, providing nitrogen (N)-containing adsorption sites. The resulting solid MPN can be employed in the adsorption system at various pH levels, starting from a pH of 2 to 10, indicating the material’s capability to withstand various environmental pH levels. The MPN has excellent adsorption efficiency for methylene blue, with an adsorption capacity of 190.81 mg/g. This efficiency was attributed to interactions such as electrostatic attraction, hydrogen bonding, dipole-dipole interactions, and n–π stacking.

Solution pH plays a critical role in the formation of MPN complexes, which directly affects their adsorption performance. Azhar et al. [36] demonstrated the impact of pH on the physicochemical properties of complexes formed between Cu^2+^ and GA, as well as their adsorption efficiency toward methylene blue and Congo red. At low pH (limited NaOH addition), insufficient hydroxide ions hinder the complete deprotonation of GA, limiting its coordination with Cu^2+^. Conversely, excessive NaOH addition can result in the oxidation of Cu^2+^, forming Cu_2_O instead of the desired complexes. Moreover, the synthesis temperature was also shown to influence the formation of the complexes. The CuGA complexes with minimal Cu_2_O impurities were achieved at a Cu:GA:NaOH molar ratio of 1:1:2.2, resulting in adsorption capacities of 124.64 mg/g for methylene blue and 344.54 mg/g for Congo red, where the molecular interaction between the adsorbent and adsorbate is shown in Figure 10c,d. In another study, Azhar et al. also demonstrated a high adsorption capacity for basic red 9 (115.08 mg/g) [41].

Isolated MPN complexes typically do not exhibit well-defined porosity. To overcome this, Lin et al. (2019) [97] developed a method to create highly ordered mesoporous MPN particles using a sacrificial templating technique. In this approach, cubosome templates were created from PS_217_-b-PEO_45_ block copolymers (PC) via a cosolvent method. An MPN precursor solution containing GA or epigallocatechin (EGCG) and FeCl_3_.6H_2_O was added to the cubosome-containing solution, and the pH was adjusted to 6.5 to drive coordination-driven assembly. The stability of Fe-ligand bonds was demonstrated under physiological conditions (pH 7.4), with Fe^3+^ ions detaching at acidic pH levels. This pH-dependent disassembly behavior is also confirmed in other works [99,100,101]. These mesoporous MPN materials exhibited exceptional protein-loading capacities (362 mg/g for glucose oxidase and 486 mg/g for horseradish peroxidase), outperforming commercial mesoporous SiO_2_ particles.

Despite the promising capabilities of GA-derived metal phenolic networks (MPNs), their adsorption potential remains underexplored, presenting an exciting avenue for future research. For instance, FeGA MPNs have demonstrated electrochemical and redox properties [34], which could be harnessed not only for metal adsorption but also for the removal of hazardous compounds through oxidation pathways. Fe-containing adsorbents have shown the ability to facilitate Fenton-like oxidation, further enhancing the degradation of organic contaminants in water. Additionally, the presence of GA in the Fenton process can enhance the efficiency of the process [102].

### 6.2. MPN for Surface Modification of Adsorbent

The surface modification of adsorbents with MPNs significantly enhances their adsorption performance by improving binding affinity and capacity. Additionally, MPN moieties impart pH-responsive behavior to the modified adsorbents. For instance, Zeng et al. [103] developed the development of Fe^3+^-GA (FeGA) MPNs that are stable at pH 5 and non-cytotoxic to mouse cells. These FeGA MPNs also exhibit photon absorption properties, making them potential photothermal guidance agents. At pH 7, FeGA undergoes decomposition due to the metabolic activity of the tested mouse cells with decomposition products efficiently excreted without causing cytotoxicity. The non-cytotoxic nature of FeGA is advantageous in applications targeting aquatic ecosystems, as it minimizes adverse effects on surrounding biota.

**Figure 11:**
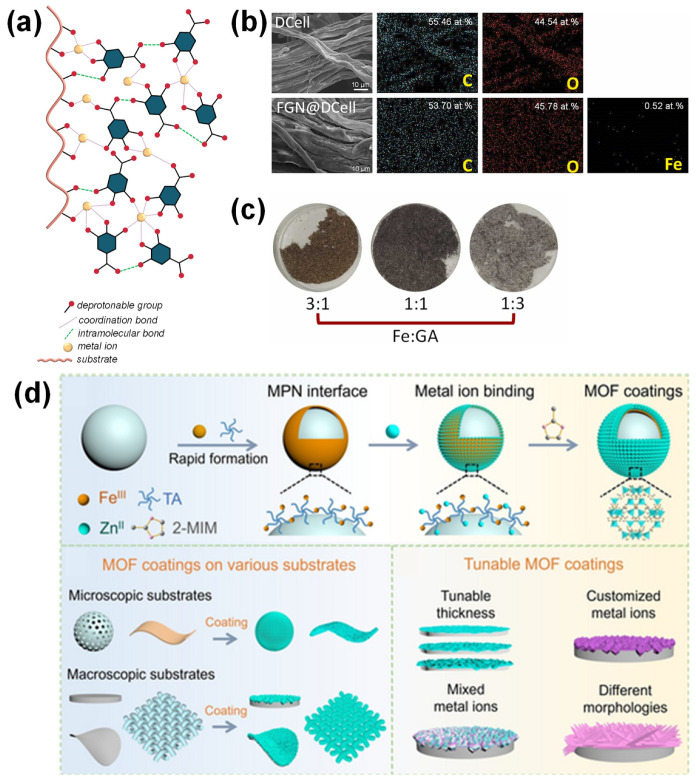
(**a**) Schematic illustration of MPN assembly on cellulose substrates. (**b**,**c**) SEM-EDX image and visual appearance of adsorbent composite prepared from coating of cellulose (DCell) with Fe-gallic acid MPN. Adapted with permission from Lunardi et al. (2024) [6], Copyright © 2023. Elsevier B.V. All rights reserved. (**d**) MPN-mediated MOF coatings: schematic representation of tunable MOF coating thickness on substrates. Adapted with permission from Wang et al. (2024) [96], Copyright © 2024 John Wiley and Sons, Inc.

Figure 11a illustrates the self-assembly process of MPN formation as a film coating on a substrate. The process begins with the deposition of metal ions onto the substrate surface. Using a substrate that contains functional groups capable of binding metal ions, such as carboxylic or amino groups, can enhance the stability of the resulting MPN coating [104]. Upon the addition of phenolic acids (e.g., GA) under suitable pH conditions, iron complexes form spontaneously, leading to the development of a supramolecular MPN coating on the substrate surface. For example, the modification of cellulose using MPN via the coordination of GA and Fe^3+^ has been successfully demonstrated by Lunardi et al. (2024) [6]. This process involved immersing the cellulose substrate in a Fe^3+^-containing solution, followed by the addition of a GA solution. MPN self-assembly was achieved by adjusting the solution pH to 8 by adding NaOH. Although the microscale size of the MPN particles hindered direct morphological observation, EDX analysis confirmed the presence of Fe, indicating successful MPN coating (Figure 11b). The modification was further evidenced by a color change on the cellulose surface, with distinct colors observed for different metal-to-ligand ratios (Figure 11c). This characteristic coloration of Fe-GA MPN has also been utilized in the preparation of dyes and pigments, such as iron gall inks [75,104]. Cellulose modified with MPN at different metal-to-ligand ratios exhibited varying removal efficiencies. The highest removal efficiency (~90%) was achieved at a 1:3 ratio, while lower efficiencies were observed at ratios of 1:1 and 3:1. The maximum adsorption capacity of MPN-modified cellulose for Cr^6+^ was 73.63 mg/g, approximately 1.6 times higher than that of unmodified cellulose. Increasing the temperature further enhanced the adsorption capacity to 116.8 mg/g.

The combination of MPNs and MOFs has become a widely adopted strategy to enhance the functionality of MOFs. FeGA MPNs, for example, offer photodynamic activity for theragnostic applications, antibacterial and antifungal properties ideal for surface coatings, and pH-responsive behavior suitable for adsorption and controlled drug release. The integration of MPNs with MOFs often involves bridging bonds, such as the Fe–O–Cu bond observed in the combination of Cu^2+^-GA (CuGA) MPN with NH_2_-MIL-88B MOF, as reported by Li et al. [105]. In this system, GA served as a chelating agent, binding Fe-metal clusters in the MOF while simultaneously coordinating with free Cu^2+^ ions. This adhesion, resulting in the formation of CuGA@NH_2_-MIL-88B, enhances the photodynamic behavior of the MOF by facilitating electron transfer from photons to the MOF through the bridging bond. Additionally, this composite demonstrated significant adsorption capability as demonstrated by a significant reduction in glutathione absorbance in the solution within 30 min of contact.

The weak adhesive nature of MOFs poses a challenge for their integration with various substrates, as direct growth of MOFs on substrate surfaces often results in particle separation due to insufficient bonding. In this context, MPNs can serve as effective bridging molecules, facilitating MOF adhesion to substrates. This process involves coating the substrate with MPNs under mild conditions, such as room temperature in aqueous solvents. The adhered MPN molecules provide active coordination sites, facilitating interactions with MOF precursors and promoting MOF growth on the substrate surface (Figure 11d). This approach has been successfully used to prepare self-standing hollow MPN@ZIF-8 structures, which function as effective H_2_/CH_4_ gas adsorbents [96]. Additionally, Luo et al. (2019) demonstrated the ease of MPN coating by applying it to a biomass-derived microporous membrane, enhancing its ability to effectively capture uranium from seawater [106].

Rahim et al. [107] demonstrated the grafting of GA onto Fe_3_O_4_ magnetic nanoparticles (FMNPs) using alkoxysilanes and the amine-containing compound 1,2-ethylenediamine (1,2-en) as mediators. In this process, the oxygen-containing group of the silane compound forms a chelation-like coordination bond with FMNPs, while the silicon moiety reacts with the ethyl group of 1,2-en via a nucleophilic-driven reaction. The amine group of 1,2-en interacts with GA through hydrogen bonding and electrostatic interactions. This composite exhibited a high adsorption capacity, binding 4.23–4.98 mmol of Fe^3+^ ions per gram of adsorbent.

Pirozzi et al. [108] explored a reverse adsorption strategy leveraging coordination chemistry between GA and metal ions. In their study, GA molecules were adsorbed onto a magnetic metal-ceramic nanocomposite by coordinating with active Lewis acid sites on the nanocomposite surface. The highest GA uptake was observed at pH 5, where the surface charge of the nanocomposite is nearly neutral, minimizing electrostatic repulsion with the moderately protonated GA. This optimal pH balanced the nanocomposite’s surface charge, GA protonation state, and Lewis acidic sites availability of for interaction.

In a recent study by Nilnit et al. [109], an in-situ strategy was developed for synthesizing a magnetic adsorbent coated with a phenolic compound. In this method, phenolics extracted from the *Hevea brasiliensis* Muell. Arg. bark was directly reacted with FeSO_4_.7H_2_O at 65 °C under sonication. The phenolic acted as ligand, chelating Fe^2+^ ions to form Fe-phenolic complexes. Sodium hydroxide was then added to the reaction mixture to precipitate Fe(OH)_2_-phenolic, which subsequently oxidized in water to produce magnetite Fe_3_O_4_-phenolic particles. This strategy resembles conventional MPN synthesis but differs in the absence of an exterior substrate, with Fe_3_O_4_ serving as both a substrate and a metal cluster. The phenolic-coated Fe_3_O_4_ selectively adsorbed tetracycline residue in honey via a solid-phase extraction process, facilitating the preconcentration of the residues before HPLC-UV analysis. The adsorbed tetracycline residues ranged from 12 to 127.2 mg/g. However, the phenolic-coated Fe_3_O_4_ particles were prone to thermal and chemical degradation, rendering them unsuitable for reuse.

While GA-MPN adsorbents offer versatile functionalities, their stability under acidic conditions remains a challenge due to protonation and the subsequent weakening of metal ligand coordination bonds, requiring further research to address this issue. One promising approach is hybridization with inorganic materials, such as metal oxides and MOFs, which can enhance structural rigidity and protect coordination sites from protonation. Additionally, complexation with metal ions that results in a higher stability constant can provide greater resistance to acidic environments. By optimizing metal ligand interactions, GA-MPNs can potentially be tailored for improved durability in low-pH applications.

### 6.3. Other MPNs-Based Adsorbent

Tannic acid (TA)—a natural biopolymer composed of eight or more gallic acid (GA) molecules—was the first phenolic compound introduced for metal-phenolic network (MPN) synthesis, as reported in the pioneering work of Ejima et al. (2013) [14]. As a structurally undefined dendritic polyphenol, it is widely used as a ligand in MPN preparation due to its abundant carboxylic and phenolic hydroxyl groups. These functional groups contribute to the complexity of determining its pKa value [110]. As pH increases, TA undergoes deprotonation, generating negatively charged ligand species that readily donate electron pairs to metal ions, forming stable complexes [83].

FeTA MPNs are particularly notable for their antifungal activity. Hou et al. demonstrated that FeTA coatings on Ag-based MOFs exhibit a synergistic antifungal effect, effectively inhibiting the growth of *Fusarium oxysporum* [111]. The FeTA coating not only provides active sites for myclobutanil loading but also enables controlled, pH-responsive release, enhancing the efficiency and precision of antifungal delivery. Furthermore, FeTA MPNs are non-cytotoxic and ecologically compatible, as evidenced by their lack of inhibitory effects on Pakchoi seed germination.

Rahim et al. (2019) explored the chelating ability of phenolic ligands for metal ion sequestration, developing a metal-phenolic sorbent (MPS) via a straightforward sol-gel process by mixing TA and Zr^4+^ in a 1:1.2 molar ratio at 85 °C for 3 min, followed by a 10 min gelation period at room temperature [112]. The resulting material exhibited nearly 100% sequestration efficiency across a broad range of metals, attributed to the active catechol and gallol groups in TA that remained unbound to Zr^4+^. Investigating GA as a ligand for MPS synthesis could provide further insights into optimizing metal sequestration performance.

MPNs also show promise as controlled-release agents for nutrients, offering a practical alternative to post-application urea removal from soil or water. Mazaheri et al. (2022) demonstrated FeTA MPN self-assembly in acetonitrile for urea coating, where complexation occurred immediately upon urea addition, leading to uniform deposition [93]. A solvent-free approach was later developed via mechanochemical grinding of MPN and urea granules, followed by aging, which enhanced complexation and coating stability [113]. The most recent work by Mazaheri et al. (2024) further improved nutrient release control by incorporating silicate into MPN-encapsulated urea [114]. Applying similar strategies to GA-based MPNs could unlock new opportunities for sustainable nutrient delivery, leveraging GA’s unique binding properties for enhanced stability and controlled release.

## 7. Conclusions and Future Perspective

### 7.1. Concluding Remark

Gallic acid (GA)-based phenolic-metal networks (GA-MPNs) have emerged as a versatile class of functional materials with broad applications in adsorption and surface modification. Their environmentally benign nature, universal adhesion properties, and tunable coordination chemistry make them highly effective for enhancing adsorbent performance. This review has provided a comprehensive overview of the fundamental chemistry governing the formation of GA-MPNs, their structural and chemical characterization, and their roles in various adsorbent architectures, including stand-alone MPNs, MPN-mediated mesoporous materials, MPN-MOF composites, and MPN-coated substrates. By consolidating recent advances in GA-based MPNs, this work contributes to the fundamental understanding required for the rational design of next-generation MPN-based adsorbents.

### 7.2. Future Research Directions

To unlock the full potential of GA-based MPNs, future research should focus on three important aspects: (1) advancing their synthesis, (2) expanding their applications in emerging fields, and (3) improving their sustainability through biomass-derived ligands.

1. Advancing MPN Synthesis and Structural Control: While GA-based MPNs have demonstrated promising adsorption properties, their amorphous nature presents challenges in precisely tuning pore size, morphology, and surface area. Future efforts should focus on:
  - Template-Assisted Synthesis: Using sacrificial templates or structure-directing agents to achieve better control over pore architecture and surface area.
  - Ligand-to-Metal Ratio Optimization: Fine-tuning coordination chemistry to enhance stability, redox properties, and adsorption performance.
  - Post-Synthesis Modifications: Functionalizing MPNs with catalytic sites, redox-active moieties, or hybrid nanomaterials to broaden their utility.
  Additionally, exploring a wider range of metal centers with tailored functionalities—such as enhanced redox activity, photodegradability, or photocatalytic properties—could expand MPN applications in energy and environmental fields.

2. Expanding Applications in Emerging Fields: Beyond adsorption and surface modification, GA-MPNs hold potential for various high-impact applications:
  - Biomedical Applications: GA’s intrinsic bioactivity, combined with metal coordination, enables potential use in antimicrobial coatings, drug delivery systems, and biosensors. Investigating MPNs as biodegradable, metal-coordinated drug carriers or bioadhesives could open new biomedical frontiers.
  - Environmental Remediation: Functionalized GA-MPNs could be engineered for targeted pollutant removal, photocatalytic degradation of contaminants, and recovery of critical metals from wastewater. Additionally, integrating MPNs with membranes or composite materials could improve their practicality in filtration technologies.
  - Energy Storage and Catalysis: MPNs with redox-active metals may serve as electrode materials in supercapacitors or electrocatalysts for water splitting and CO₂ reduction.
3. Sustainable Development Using Biomass-Derived Ligands: A promising avenue for cost-effective and eco-friendly MPN development is the use of crude biomass extracts as sources of phenolic ligand instead of purified GA. However, key challenges remain:
  - Extraction Optimization: Developing efficient, scalable methods to obtain high-phenolic-content extracts with minimal impurities.
  - Ligand Purity Control: Understanding the impact of natural extract variability on MPN formation and performance.
  - Complexation Efficiency: Investigating how mixed phenolic compounds in crude extracts influence coordination chemistry and material stability.

By addressing these challenges, GA-based MPNs could become a sustainable and economically viable alternative for adsorption, catalysis, and biomedicine. Continued interdisciplinary research integrating chemistry, materials science, and engineering will be essential to fully realize their potential in both scientific and industrial domains.

## Figures and Tables

**Table 1:** Ionization or dissociation constant of the gallic acid.

Condition	pKa_1_	pKa_2_	pKa_3_	pKa_4_	Ref.
*I* = 0.2 M ^1^ and *T* = 25 °C	4.22	8.69	11.19		[60]
*I* = 0.1 M ^1^ and *T* = 25 °C	4.4	8.6	11.2	12	[24]
*I* = 0.1 M NaNO_3_ and *T* = 25 °C	4.10	8.38			[17]
*I* = 0.1 M NaNO_3_ and *T* = 25 °C	4.12	8.32			[61]
*I* = 0.1 M KCl and *T* = 25 °C	3.75	7.50	9.50	10.50	[62]

^1^ Salt source undefined.

**Table 2:** Stability constant (log*K*) of complexes between GA and transition metal ions.

Metal	Complex Species M*_p_*L*_q_*		Condition	log*K*	Ref.
	*p*	*q*			
Cu^2+^	1	1	*I* = 0.1 M NaNO_3_, *T* = 25 °C	9.75	[61]
			*I* = 1 N NaNO_3_, *T* = 27 °C	9.80	[26]
	1	2	*I* = 0.1 M NaNO_3_, *T* = 25 °C	6.75	[61]
Zn^2+^	1	1	*I* = 0.1 M NaNO_3_, *T* = 25 °C	8.56	[61]
			*I* = 1 N NaNO_3_, *T* = 27 °C	7.98	[26]
	1	2	*I* = 0.1 M NaNO_3_, *T* = 25 °C	5.83	[61]
	2	1	*I* = 0.1 M CaCl_2_, *T* = 25 °C, pH = 8	11.38	[24]
Ni^2+^	1	1	*I* = 0.1 M NaNO_3_, *T* = 25 °C	8.00	[61]
			*I* = 1 N NaNO_3_, *T* = 27 °C	6.74	[26]
	1	2	*I* = 0.1 M NaNO_3_, *T* = 25 °C	5.50	[61]
Fe^3+^	1	1	*I* = 0.1 M NaNO_3_, *T* = 25 °C	14.73	[17]
			*I* = 1 N NaNO_3_, *T* = 27 °C	10.98	[26]
	1	2	*I* = 0.1 M NaNO_3_, *T* = 25 °C	11.93	[61]
Co^2+^	1	1	*I* = 0.1 M NaNO_3_, *T* = 25 °C	7.25	[61]
			*I* = 1 N NaNO_3_, *T* = 27 °C	7.13	[26]
	1	2	*I* = 0.1 M NaNO_3_, *T* = 25 °C	4.75	[61]

## Data Availability

No primary research results have been included and no new data were generated or analyzed as part of this review.

## Supplementary Materials

The following supporting information can be downloaded at: https://www.mdpi.com/article/10.3390/molecules30061218/s1, Table S1: Gallic acid-based metal complexes; Table S2: Hard-Soft Acid-Base List; Table S3: Data point of titration curves of solution containing gallic acid, Fe^3+^, and glycine at 0.1 M ionic strength; Table S4: HySS2009-Concentration table. References [115,116,117,118,119] are cited in the supplementary materials.

## Abbreviations

The following abbreviations are used in this manuscript:

MPNs	Metal-phenolic networks
MOFs	Metal-organic frameworks
GA	Gallic acid
TA	Tannic acid
HSAB	Hard-Soft Acid-Base
IWS	Irving Williams Series
SEM	Scanning electron microscopy
EDX	Energy dispersive X-ray
SERS	Surface-Enhanced Raman Spectroscopy
FTIR	Fourier transform infrared spectroscopy
EPR	Electron Paramagnetic Resonance
NMR	Nuclear Magnetic Resonance
TEM	Transmission electron microscopy
HPLC	High performance liquid chromatography
DFT	Density functional theory
SSA	Surface specific area
PC	PS_217_-b-PEO_45_ block copolymers
EGCG	Epigallocatechin
en	Ethylenediamine
FMNPs	Fe_3_O_4_ magnetic nanoparticles
LMCT	Ligand-to-metal charge transfer
