# Supplementary material for: Unlocking the Potential of Gallic Acid-Based Metal Phenolic Networks for Innovative Adsorbent Design
Source: Molecules. 2025 Mar 8;30(6):1218. doi: 10.3390/molecules30061218 (PMC11945622; doi:10.3390/molecules30061218)
Supplement: Supplementary file 1 [file molecules-3453376-supplementary.pdf › molecules-3453376-supplementary.pdf]

Supplementary Information (SI)

Unlocking the Potential of Gallic Acid-Based Metal-Phenolic Networks for Innovative Adsorbent Design

Shella Permatasari Santoso, Artik Elisa Angkawijaya, Kuan-Chen Cheng, Shin-Ping Lin, Hsien-Yi Hsu, Chang-Wei Hsieh, Astrid Rahmawati, Osamu Shimomura, and Suryadi Ismadji

Table S1. Gallic acid-based metal complexes

Year	Category	Purpose/ Targeted Use	Metal ion(s)	Publication title	Ref.
			Metal complexes		
1954	Coordination chemistry	Analysis	Ti^{4+}	Studies of gallic acid complexes with metals and their analytical applications - Part I. Spectrophotometric investigation	[1]
1956	Coordination chemistry	Analysis	Mo^{2+}	Studies of gallic acid complexes with metals and their analytical applications – Part II. A Spectrophotometric Study of molybdenum complexes	[2]
1956	Coordination chemistry	Analysis	Ti^{4+}	Studies of gallic acid complexes with metals and their analytical applications - Part III. Spectrophotometric estimation of gallic acid	[3]
1976	Coordination chemistry	Analysis	Various	Polarography of metal-gallic complexes	[4]
1982	Coordination chemistry	Analysis	Fe^{2+}, Fe^{3+}	Interactions of Iron(II) and Iron(III) with Gallic Acid and its Homologues: a Potentiormetric and Spectrophotometric Study	[5]
1985	Coordination chemistry	Analysis	Ca^{2+}, Mg^{2+}, Zn^{2+}	Stability constant of calcium, magnesium and zinc gallate using a divalent ion-selective electrode	[6]
2008	Coordination chemistry	Analysis	Fe^{3+}	Iron Complexation Studies of Gallic Acid	[7]
2012	Computational study	Analysis	Fe^{3+}	Iron complexes with gallic acid: a computational study on coordination compounds of interest for the preservation of cultural heritage	[8]
2014	Coordination chemistry	Spectroscopic study	Fe^{3+}	Spectroscopic studies on gallic acid and its azo derivatives and their iron(III) complexes	[9]
2015	Material	Sensor development	Fe_2O_3	An electrochemical sensor for gallic acid based on Fe_2O_3/electro-reduced graphene oxide composite: Estimation for the antioxidant capacity index of wines	[10]
2015	Therapy	Cancer treatment	Au	Gallic acid enhancement of gold nanoparticle anticancer activity in cervical cancer cells	[11]
2016	Coordination chemistry	Formation constant	Transition metals	Protonation Equilibria of Gallic Acid (GA) and Stability Constants of Its Complexes with Transition Metal Ions in Aqueous Media	[12]
2018	Therapy	Tumor treatment	Fe^{3+}	Functional-Protein-Assisted Fabrication of Fe–Gallic Acid Coordination Polymer Nanonetworks for Localized Photothermal Therapy	[13]
2019	Therapy	Neurodegenerative disease	Mg^{2+}	Gallic acid: A naturally occurring bifunctional inhibitor of amyloid and metal induced aggregation with possible implication in metal-based therapy	[14]
2021	Therapy	Tumor treatment	Au	Gallic acid-gold nanoparticles enhance radiation-induced cell death of human glioma U251 cells	[15]
2021	Coordination chemistry	Formation constant	Fe^{2+}	Dependence of the Fe(II)-Gallic Acid Coordination Compound Formation Constant on the pH	[16]

Table S1. Gallic acid-based metal complexes (continued)

Year	Category	Purpose/ Targeted Use	Metal ion(s)	Publication title	Ref.
Metal complexes					
2022	Coordination chemistry	Characteri-zation	Ca^{2+}, Cu^{2+}, Zn^{3+}, Cr^{3+}, Se^{4+}	Synthesis, spectroscopic characterizations, conductometric titration and investigation of potent antioxidant activities of gallic acid complexes with Ca (II), Cu (II), Zn(III), Cr(III) and Se (IV) metal ions	[17]
2023	Material	Sensor	Al	The Application of Alumina for Electroanalytical Determination of Gallic Acid	[18]
2023	Coordination chemistry	Characteri-zation	Eu^{3+}	Europium(III) and gallic acid ligand coordination compounds: synthesis, characterization, photophysical processes, and optimization of luminescent properties	[19]
2024	Coordination chemistry	Formation constant	La^{3+}, Sm^{3+}, Th^{4+}, UO_2^{2+}	Complexation of gallic acid involving La^{3+}, Sm^{3+}, Th^{4+} and UO_2^{2+} ions in aqueous solution by potentiometry at various temperatures	[20]
2024	Coordination chemistry	Formation constant	Ca^{2+}	Complexation of gallic acid with calcium: electrochemical, potentiometric, and UV-VIS studies	[21]
2025	Therapy	Tumor treatment	Ca^{2+}	Photothermal switch by gallic acid-calcium grafts synthesized by coordination chemistry for sequential treatment of bone tumor and regeneration	[22]
Metal-Phenolic Networks					
2018	Material	Drug delivery	Fe^{3+}	Electrochemical Behavior and Redox-Dependent Disassembly of Gallic Acid/Fe^{III} Metal–Phenolic Networks	[23]
2021	Material	Adsorption	Cu^{2+}	One-step synthesis of nitrogen-grafted copper-gallic acid for enhanced methylene blue removal	[24]
2022	Material	Coating	Fe^{3+}	Metal-phenolic networks as tuneable spore coat mimetics	[25]
Metal-Organic Frameworks					
2019	Therapy	Cancer treatment	Cu^{2+}	Copper-Gallic Acid Nanoscale Metal–Organic Framework for Combined Drug Delivery and Photodynamic Therapy	[26]
2019	Material	Adsorption	Zr-MOF	Synergistic Effect of Metal–Organic Framework/Gallic Acid in Enhanced Laser Desorption/Ionization Mass Spectrometry	[27]
2020	Material	Adsorption	Cu^{2+}	Aqueous synthesis of highly adsorptive copper–gallic acid metal–organic framework	[28]
2023	Material	Drug delivery	Ni^{2+}	Nickel-gallate metal–organic framework as an efficient antimicrobial and anticancer agent: in vitro study	[29]
2023	Material	Adsorption	Cu^{2+}	Green synthesis optimization with artificial intelligence studies of copper–gallic acid metal–organic framework and its application in dye removal from wastewater	[30]
2024	Material	Drug delivery	Cu^{2+}	In-situ synthesis of copper-gallic acid metal–organic framework into the gentamicin-loaded chitosan hydrogel bead: A synergistic enhancement of antibacterial properties	[31]
2025	Therapy	Tumor treatment	Fe^{3+}	A glutathione-activated bismuth-gallic acid metal-organic framework nano-prodrug for enhanced sonodynamic therapy of breast tumor	[32]

Table S2. Hard-Soft Acid-Base List

Lewis Acids		Lewis Bases
H^+, Li^+, Na^+, K^+<br>Be^{2+}, Mg^{2+}, Ca^{2+}, Cr^{2+}<br>Fe^{3+}, Cr^{3+}, Al^{3+}, SO_3, BF_3	Hard	OH^-, F^-, H_2O<br>$CH_3CO_2^-$, PO_4^{3-}, SO_4^{2-}<br>Cl^-, CO_3^{2-}, ClO_4^-, NO_3^-<br>R-OH, R-O^-, R_2O<br>NH_3, R-NH_2, N_2H_4
Fe^{2+}, Co^{2+}, Ni^{2+}, Cu^{2+}, Zn^{2+}, Pb^{2+}	Borderline	$C_6H_5NH_2$, C_5H_5N, N_3^-, Br-, NO_2^-, SO_3^{2-}, N_2
Cu^+, Au^+, Ag^+<br>TI^+, Hg_2^{2+}, Pd^{2+}<br>Cd^{2+}, Pt^{2+}, Hg^{2+}<br>BH_3	Soft	R_2S, R-SH, R-S^-<br>I^-, SCN^-, $S_2O_3^{2-}$<br>R_3-P, R_3-As, $(R\text{-}O)_3P$<br>CN^-, R-NC, CO<br>H^-, R^-

Table S3. Data point of titration curves of solution containing gallic acid, Fe3+, and glycine at 0.1 M ionic strength

Curve (i)		Curve (ii)		Curve (iii)		Curve (iv)		Curve (v)		Curve (vi)	
NaOH (mL)	pH	NaOH (mL)	pH	NaOH (mL)	pH	NaOH (mL)	pH	NaOH (mL)	pH	NaOH (mL)	pH
0.992754	3.21522	1	3.21522	1	3.18147	1	3.21519	1	3.01259	1	2.99578
1.0942	3.38351	1.0942	3.40039	1.0942	3.40039	1.10145	3.41723	1.8913	3.19401	2.03623	3.19332
1.18841	3.61934	1.18116	3.6025	1.17391	3.58566	1.18116	3.6025	2.27536	3.39469	2.49275	3.39365
1.22464	3.8217	1.22464	3.78795	1.23188	3.80479	1.4058	3.78707	2.48551	3.59621	2.73913	3.56123
1.26087	3.9903	1.26087	3.9903	1.27536	4.04087	1.55797	3.95512	2.64493	3.79798	2.94928	3.77863
1.26812	4.24343	1.27536	4.17589	1.27536	4.19276	1.67391	4.19084	2.71739	4.00016	3.0942	3.98146
1.2971	4.41207	1.2971	4.39519	1.30435	4.41203	1.78261	4.40973	2.75362	4.20251	3.27536	4.18312
1.31884	4.66513	1.31159	4.5639	1.31884	4.59762	1.83333	4.59514	2.78986	4.40487	3.63768	4.36703
1.32609	4.90138	1.32609	4.80012	1.34058	4.80005	1.86232	4.78065	2.81159	4.6073	3.94928	4.58493
1.34783	5.23883	1.34783	4.98566	1.34783	4.96879	1.86957	5.00003	2.84783	4.80965		
1.36957	5.61003	1.35507	5.18816	1.37681	5.20493	1.9058	5.20238	2.86957	4.9952		
1.36957	5.81256	1.35507	5.39069	1.3913	5.44115	1.92029	5.4386	2.9058	5.23132		
1.38406	6.01503	1.36957	5.57628	1.41304	5.6267	1.92029	5.60738	2.94203	5.4168		
1.39855	6.23437	1.37681	5.77877	1.43478	5.846	1.94928	5.80977	2.96377	5.61922		
1.42754	6.41988	1.3913	5.99811	1.43478	6.03166	1.96377	6.01223	2.96377	5.80488		
1.43478	6.6055	1.4058	6.20057	1.44203	6.25103	1.97101	6.21473	2.98551	5.99043		
1.44203	6.808	1.42754	6.403	1.47101	6.38591	1.98551	6.38343	3	6.17601		
1.45652	7.01046	1.43478	6.58862	1.5	6.60518	2	6.5859	3.02899	6.39528		
1.45652	7.21299	1.44928	6.79108	1.52899	6.82445	2.00725	6.82215	3.05072	6.58083		
1.46377	7.43236	1.44928	7.01049	1.56522	7.00993	2.02174	6.99085	3.06522	6.78329		
1.46377	7.60114	1.44928	7.19615	1.63043	7.21215	2.02174	7.19339	3.08696	7.00259		
1.47826	7.82048	1.45652	7.39864	1.68116	7.39756	2.03623	7.39585	3.0942	7.20509		
1.48551	7.98922	1.47101	7.58423	1.75362	7.59974	2.04348	7.58147	3.1087	7.40755		
1.49275	8.17484	1.47101	7.76988	1.81159	7.80199	2.05797	7.80081	3.13043	7.57622		
1.50725	8.41106	1.47826	7.98925	1.85507	8.00432	2.06522	8.0033	3.15217	7.77865		
1.50725	8.59671	1.48551	8.19175	1.91304	8.22345	2.07246	8.17204				

Table S3. Data point of titration curves of solution containing gallic acid, Fe3+, and glycine at 0.1 M ionic strength (continued)

Curve (i)		Curve (ii)		Curve (iii)		Curve (iv)		Curve (v)		Curve (vi)	
NaOH (mL)	pH	NaOH (mL)	pH	NaOH (mL)	pH	NaOH (mL)	pH	NaOH (mL)	pH	NaOH (mL)	pH
1.51449	8.81608	1.54348	8.37713	1.94203	8.42584	2.07971	8.4083				
1.52899	9.00167	1.55797	8.59647	1.97826	8.59444	2.0942	8.62763				
1.52899	9.2042	1.58696	8.78198	2	8.79687	2.0942	8.81329				
1.53623	9.4067	1.6087	8.98441	2.05072	9.03291						
1.55797	9.60912	1.67391	9.18662	2.0942	9.20147						
1.58696	9.82839	1.74638	9.38881	2.14493	9.42064						
1.63043	9.99696	1.78986	9.62488	2.21014	9.62286						
1.68116	10.2161	1.89855	9.77626	2.28261	9.79128						
1.78261	10.4013	2	9.96142	2.39855	10.0101						
		2.10145	10.2141	2.5	10.1953						
		2.26812	10.4158	2.65217	10.3802						

Table S4. HySS2009 - Concentration table. Created at 11/26/2024 2:09:11 PM

Input parameter

Species	log beta
GAH	12
GAH2	23.2
GAH3	31.8
GAH4	36.2
FeGA	14.73
FeGA2	26.66
H-1	-13.77

	**total Fe**	**total GA**	**total H**	**free Fe**	**free GA**	**p(H)**	**GAH**	**GAH2**	**GAH3**	**GAH4**	**FeGA**	**FeGA2**	**OH**
1	2.00E-01	2.00E-01	8.09E-01	2.00E-01	1.26E-29	2	1.26E-19	1.99E-10	7.93E-04	1.99E-01	1.35E-15	1.44E-32	1.70E-12
2	2.00E-01	2.00E-01	8.07E-01	2.00E-01	3.79E-29	2.12	2.88E-19	3.46E-10	1.04E-03	1.99E-01	4.07E-15	1.31E-31	2.24E-12
3	2.00E-01	2.00E-01	8.04E-01	2.00E-01	1.14E-28	2.24	6.58E-19	6.00E-10	1.37E-03	1.99E-01	1.23E-14	1.19E-30	2.95E-12
4	2.00E-01	2.00E-01	8.03E-01	2.00E-01	3.44E-28	2.36	1.50E-18	1.04E-09	1.81E-03	1.98E-01	3.70E-14	1.08E-29	3.89E-12
5	2.00E-01	2.00E-01	8.01E-01	2.00E-01	1.04E-27	2.48	3.43E-18	1.80E-09	2.38E-03	1.98E-01	1.11E-13	9.83E-29	5.13E-12
6	2.00E-01	2.00E-01	7.99E-01	2.00E-01	3.12E-27	2.6	7.84E-18	3.12E-09	3.12E-03	1.97E-01	3.35E-13	8.90E-28	6.76E-12
7	2.00E-01	2.00E-01	7.98E-01	2.00E-01	9.38E-27	2.72	1.79E-17	5.40E-09	4.09E-03	1.96E-01	1.01E-12	8.04E-27	8.91E-12
8	2.00E-01	2.00E-01	7.96E-01	2.00E-01	2.81E-26	2.84	4.07E-17	9.32E-09	5.36E-03	1.95E-01	3.02E-12	7.24E-26	1.18E-11
9	2.00E-01	2.00E-01	7.94E-01	2.00E-01	8.42E-26	2.96	9.24E-17	1.61E-08	7.01E-03	1.93E-01	9.05E-12	6.49E-25	1.55E-11
10	2.00E-01	2.00E-01	7.92E-01	2.00E-01	2.52E-25	3.08	2.09E-16	2.76E-08	9.14E-03	1.91E-01	2.70E-11	5.79E-24	2.04E-11
11	2.00E-01	2.00E-01	7.89E-01	2.00E-01	7.49E-25	3.2	4.73E-16	4.73E-08	1.19E-02	1.88E-01	8.04E-11	5.13E-23	2.69E-11
12	2.00E-01	2.00E-01	7.85E-01	2.00E-01	2.22E-24	3.32	1.06E-15	8.06E-08	1.54E-02	1.85E-01	2.38E-10	4.51E-22	3.55E-11
13	2.00E-01	2.00E-01	7.81E-01	2.00E-01	6.54E-24	3.44	2.38E-15	1.37E-07	1.98E-02	1.80E-01	7.03E-10	3.92E-21	4.68E-11
14	2.00E-01	2.00E-01	7.75E-01	2.00E-01	1.92E-23	3.56	5.28E-15	2.30E-07	2.53E-02	1.75E-01	2.06E-09	3.36E-20	6.17E-11
15	2.00E-01	2.00E-01	7.68E-01	2.00E-01	5.56E-23	3.68	1.16E-14	3.85E-07	3.20E-02	1.68E-01	5.98E-09	2.83E-19	8.13E-11
16	2.00E-01	2.00E-01	7.60E-01	2.00E-01	1.60E-22	3.8	2.53E-14	6.36E-07	4.02E-02	1.60E-01	1.72E-08	2.34E-18	1.07E-10
17	2.00E-01	2.00E-01	7.50E-01	2.00E-01	4.54E-22	3.92	5.46E-14	1.04E-06	4.98E-02	1.50E-01	4.87E-08	1.88E-17	1.41E-10

Table S4. HySS2009 - Concentration table. (continued)

18	2.00E-01	2.00E-01	7.39E-01	2.00E-01	1.27E-21	4.04	1.16E-13	1.67E-06	6.08E-02	1.39E-01	1.36E-07	1.47E-16	1.86E-10
19	2.00E-01	2.00E-01	7.27E-01	2.00E-01	3.50E-21	4.16	2.42E-13	2.65E-06	7.31E-02	1.27E-01	3.76E-07	1.12E-15	2.46E-10
20	2.00E-01	2.00E-01	7.14E-01	2.00E-01	9.46E-21	4.28	4.96E-13	4.13E-06	8.63E-02	1.14E-01	1.02E-06	8.18E-15	3.24E-10
21	2.00E-01	2.00E-01	7.00E-01	2.00E-01	2.51E-20	4.4	1.00E-12	6.31E-06	1.00E-01	1.00E-01	2.70E-06	5.77E-14	4.27E-10
22	2.00E-01	2.00E-01	6.86E-01	2.00E-01	6.54E-20	4.52	1.98E-12	9.46E-06	1.14E-01	8.63E-02	7.03E-06	3.92E-13	5.62E-10
23	2.00E-01	2.00E-01	6.73E-01	2.00E-01	1.67E-19	4.64	3.83E-12	1.39E-05	1.27E-01	7.30E-02	1.80E-05	2.56E-12	7.41E-10
24	2.00E-01	2.00E-01	6.61E-01	2.00E-01	4.20E-19	4.76	7.30E-12	2.01E-05	1.39E-01	6.08E-02	4.51E-05	1.62E-11	9.77E-10
25	2.00E-01	2.00E-01	6.49E-01	2.00E-01	1.04E-18	4.88	1.37E-11	2.86E-05	1.50E-01	4.97E-02	1.12E-04	9.86E-11	1.29E-09
26	2.00E-01	2.00E-01	6.39E-01	2.00E-01	2.53E-18	5	2.53E-11	4.01E-05	1.60E-01	4.01E-02	2.71E-04	5.84E-10	1.70E-09
27	2.00E-01	2.00E-01	6.30E-01	1.99E-01	6.08E-18	5.12	4.61E-11	5.54E-05	1.67E-01	3.19E-02	6.51E-04	3.37E-09	2.24E-09
28	2.00E-01	2.00E-01	6.20E-01	1.99E-01	1.44E-17	5.24	8.30E-11	7.57E-05	1.73E-01	2.51E-02	1.54E-03	1.89E-08	2.95E-09
29	2.00E-01	2.00E-01	6.09E-01	1.96E-01	3.37E-17	5.36	1.47E-10	1.02E-04	1.77E-01	1.94E-02	3.56E-03	1.02E-07	3.89E-09
30	2.00E-01	2.00E-01	5.91E-01	1.92E-01	7.73E-17	5.48	2.56E-10	1.34E-04	1.77E-01	1.47E-02	7.98E-03	5.25E-07	5.13E-09
31	2.00E-01	2.00E-01	5.60E-01	1.83E-01	1.72E-16	5.6	4.32E-10	1.72E-04	1.72E-01	1.09E-02	1.69E-02	2.48E-06	6.76E-09
32	2.00E-01	2.00E-01	5.09E-01	1.67E-01	3.65E-16	5.72	6.96E-10	2.10E-04	1.59E-01	7.63E-03	3.28E-02	1.02E-05	8.91E-09
33	2.00E-01	2.00E-01	4.36E-01	1.44E-01	7.27E-16	5.84	1.05E-09	2.41E-04	1.39E-01	5.03E-03	5.62E-02	3.47E-05	1.18E-08
34	2.00E-01	2.00E-01	3.50E-01	1.16E-01	1.35E-15	5.96	1.48E-09	2.58E-04	1.12E-01	3.10E-03	8.41E-02	9.67E-05	1.55E-08
35	2.00E-01	2.00E-01	2.65E-01	8.81E-02	2.36E-15	6.08	1.97E-09	2.59E-04	8.58E-02	1.79E-03	1.12E-01	2.25E-04	2.04E-08
36	2.00E-01	2.00E-01	1.92E-01	6.41E-02	3.94E-15	6.2	2.48E-09	2.48E-04	6.24E-02	9.89E-04	1.36E-01	4.54E-04	2.69E-08
37	2.00E-01	2.00E-01	1.34E-01	4.53E-02	6.32E-15	6.32	3.03E-09	2.30E-04	4.37E-02	5.26E-04	1.54E-01	8.28E-04	3.55E-08
38	2.00E-01	2.00E-01	9.07E-02	3.16E-02	9.84E-15	6.44	3.57E-09	2.06E-04	2.97E-02	2.71E-04	1.67E-01	1.40E-03	4.68E-08
39	2.00E-01	2.00E-01	5.95E-02	2.21E-02	1.48E-14	6.56	4.08E-09	1.78E-04	1.95E-02	1.35E-04	1.76E-01	2.22E-03	6.17E-08
40	2.00E-01	2.00E-01	3.74E-02	1.58E-02	2.14E-14	6.68	4.46E-09	1.48E-04	1.23E-02	6.45E-05	1.81E-01	3.29E-03	8.13E-08
41	2.00E-01	2.00E-01	2.20E-02	1.19E-02	2.88E-14	6.8	4.56E-09	1.15E-04	7.23E-03	2.88E-05	1.84E-01	4.50E-03	1.07E-07
42	2.00E-01	2.00E-01	1.20E-02	9.63E-03	3.57E-14	6.92	4.30E-09	8.19E-05	3.92E-03	1.18E-05	1.85E-01	5.62E-03	1.41E-07
43	2.00E-01	2.00E-01	5.99E-03	8.44E-03	4.08E-14	7.04	3.72E-09	5.38E-05	1.95E-03	4.48E-06	1.85E-01	6.43E-03	1.86E-07
44	2.00E-01	2.00E-01	2.82E-03	7.87E-03	4.39E-14	7.16	3.03E-09	3.33E-05	9.16E-04	1.59E-06	1.85E-01	6.91E-03	2.46E-07

Table S4. HySS2009 - Concentration table. (continued)

45	2.00E-01	2.00E-01	1.28E-03	7.60E-03	4.54E-14	7.28	2.38E-09	1.98E-05	4.14E-04	5.46E-07	1.85E-01	7.16E-03	3.24E-07
46	2.00E-01	2.00E-01	5.75E-04	7.47E-03	4.62E-14	7.4	1.84E-09	1.16E-05	1.84E-04	1.84E-07	1.85E-01	7.28E-03	4.27E-07
47	2.00E-01	2.00E-01	2.56E-04	7.42E-03	4.65E-14	7.52	1.40E-09	6.72E-06	8.08E-05	6.13E-08	1.85E-01	7.33E-03	5.62E-07
48	2.00E-01	2.00E-01	1.13E-04	7.40E-03	4.67E-14	7.64	1.07E-09	3.88E-06	3.54E-05	2.04E-08	1.85E-01	7.36E-03	7.41E-07
49	2.00E-01	2.00E-01	5.00E-05	7.38E-03	4.67E-14	7.76	8.12E-10	2.24E-06	1.55E-05	6.75E-09	1.85E-01	7.37E-03	9.77E-07
50	2.00E-01	2.00E-01	2.16E-05	7.38E-03	4.68E-14	7.88	6.16E-10	1.29E-06	6.76E-06	2.24E-09	1.85E-01	7.37E-03	1.29E-06
51	2.00E-01	2.00E-01	8.65E-06	7.38E-03	4.68E-14	8	4.68E-10	7.41E-07	2.95E-06	7.41E-10	1.85E-01	7.37E-03	1.70E-06
52	2.00E-01	2.00E-01	2.49E-06	7.38E-03	4.68E-14	8.12	3.55E-10	4.27E-07	1.29E-06	2.45E-10	1.85E-01	7.37E-03	2.24E-06
53	2.00E-01	2.00E-01	-7.67E-07	7.38E-03	4.68E-14	8.24	2.69E-10	2.46E-07	5.62E-07	8.13E-11	1.85E-01	7.38E-03	2.95E-06
54	2.00E-01	2.00E-01	-2.87E-06	7.38E-03	4.68E-14	8.36	2.04E-10	1.41E-07	2.46E-07	2.69E-11	1.85E-01	7.38E-03	3.89E-06
55	2.00E-01	2.00E-01	-4.64E-06	7.38E-03	4.68E-14	8.48	1.55E-10	8.13E-08	1.07E-07	8.91E-12	1.85E-01	7.38E-03	5.13E-06
56	2.00E-01	2.00E-01	-6.52E-06	7.38E-03	4.68E-14	8.6	1.18E-10	4.68E-08	4.68E-08	2.95E-12	1.85E-01	7.38E-03	6.76E-06
57	2.00E-01	2.00E-01	-8.80E-06	7.38E-03	4.68E-14	8.72	8.91E-11	2.69E-08	2.04E-08	9.77E-13	1.85E-01	7.38E-03	8.91E-06
58	2.00E-01	2.00E-01	-1.17E-05	7.38E-03	4.68E-14	8.84	6.76E-11	1.55E-08	8.91E-09	3.24E-13	1.85E-01	7.38E-03	1.18E-05
59	2.00E-01	2.00E-01	-1.55E-05	7.38E-03	4.68E-14	8.96	5.13E-11	8.91E-09	3.89E-09	1.07E-13	1.85E-01	7.38E-03	1.55E-05
60	2.00E-01	2.00E-01	-2.04E-05	7.38E-03	4.68E-14	9.08	3.89E-11	5.13E-09	1.70E-09	3.55E-14	1.85E-01	7.38E-03	2.04E-05
61	2.00E-01	2.00E-01	-2.69E-05	7.38E-03	4.68E-14	9.2	2.95E-11	2.95E-09	7.41E-10	1.18E-14	1.85E-01	7.38E-03	2.69E-05
62	2.00E-01	2.00E-01	-3.55E-05	7.38E-03	4.68E-14	9.32	2.24E-11	1.70E-09	3.24E-10	3.89E-15	1.85E-01	7.38E-03	3.55E-05
63	2.00E-01	2.00E-01	-4.68E-05	7.38E-03	4.68E-14	9.44	1.70E-11	9.77E-10	1.41E-10	1.29E-15	1.85E-01	7.38E-03	4.68E-05
64	2.00E-01	2.00E-01	-6.17E-05	7.38E-03	4.68E-14	9.56	1.29E-11	5.62E-10	6.17E-11	4.27E-16	1.85E-01	7.38E-03	6.17E-05
65	2.00E-01	2.00E-01	-8.13E-05	7.38E-03	4.68E-14	9.68	9.77E-12	3.24E-10	2.69E-11	1.41E-16	1.85E-01	7.38E-03	8.13E-05
66	2.00E-01	2.00E-01	-1.07E-04	7.38E-03	4.68E-14	9.8	7.41E-12	1.86E-10	1.18E-11	4.68E-17	1.85E-01	7.38E-03	1.07E-04
67	2.00E-01	2.00E-01	-1.41E-04	7.38E-03	4.68E-14	9.92	5.62E-12	1.07E-10	5.13E-12	1.55E-17	1.85E-01	7.38E-03	1.41E-04
68	2.00E-01	2.00E-01	-1.86E-04	7.38E-03	4.68E-14	10.04	4.27E-12	6.17E-11	2.24E-12	5.13E-18	1.85E-01	7.38E-03	1.86E-04
69	2.00E-01	2.00E-01	-2.46E-04	7.38E-03	4.68E-14	10.16	3.24E-12	3.55E-11	9.77E-13	1.70E-18	1.85E-01	7.38E-03	2.46E-04
70	2.00E-01	2.00E-01	-3.24E-04	7.38E-03	4.68E-14	10.28	2.46E-12	2.04E-11	4.27E-13	5.62E-19	1.85E-01	7.38E-03	3.24E-04
71	2.00E-01	2.00E-01	-4.27E-04	7.38E-03	4.68E-14	10.4	1.86E-12	1.18E-11	1.86E-13	1.86E-19	1.85E-01	7.38E-03	4.27E-04

Table S4. HySS2009 - Concentration table. (continued)

72	2.00E-01	2.00E-01	-5.62E-04	7.38E-03	4.68E-14	10.52	1.41E-12	6.76E-12	8.13E-14	6.17E-20	1.85E-01	7.38E-03	5.62E-04
73	2.00E-01	2.00E-01	-7.41E-04	7.38E-03	4.68E-14	10.64	1.07E-12	3.89E-12	3.55E-14	2.04E-20	1.85E-01	7.38E-03	7.41E-04
74	2.00E-01	2.00E-01	-9.77E-04	7.38E-03	4.68E-14	10.76	8.13E-13	2.24E-12	1.55E-14	6.76E-21	1.85E-01	7.38E-03	9.77E-04
75	2.00E-01	2.00E-01	-1.29E-03	7.38E-03	4.68E-14	10.88	6.17E-13	1.29E-12	6.76E-15	2.24E-21	1.85E-01	7.38E-03	1.29E-03
76	2.00E-01	2.00E-01	-1.70E-03	7.38E-03	4.68E-14	11	4.68E-13	7.41E-13	2.95E-15	7.41E-22	1.85E-01	7.38E-03	1.70E-03
77	2.00E-01	2.00E-01	-2.24E-03	7.38E-03	4.68E-14	11.12	3.55E-13	4.27E-13	1.29E-15	2.46E-22	1.85E-01	7.38E-03	2.24E-03
78	2.00E-01	2.00E-01	-2.95E-03	7.38E-03	4.68E-14	11.24	2.69E-13	2.46E-13	5.62E-16	8.13E-23	1.85E-01	7.38E-03	2.95E-03
79	2.00E-01	2.00E-01	-3.89E-03	7.38E-03	4.68E-14	11.36	2.04E-13	1.41E-13	2.46E-16	2.69E-23	1.85E-01	7.38E-03	3.89E-03
80	2.00E-01	2.00E-01	-5.13E-03	7.38E-03	4.68E-14	11.48	1.55E-13	8.13E-14	1.07E-16	8.91E-24	1.85E-01	7.38E-03	5.13E-03
81	2.00E-01	2.00E-01	-6.76E-03	7.38E-03	4.68E-14	11.6	1.18E-13	4.68E-14	4.68E-17	2.95E-24	1.85E-01	7.38E-03	6.76E-03
82	2.00E-01	2.00E-01	-8.91E-03	7.38E-03	4.68E-14	11.72	8.91E-14	2.69E-14	2.04E-17	9.77E-25	1.85E-01	7.38E-03	8.91E-03
83	2.00E-01	2.00E-01	-1.18E-02	7.38E-03	4.68E-14	11.84	6.76E-14	1.55E-14	8.91E-18	3.24E-25	1.85E-01	7.38E-03	1.18E-02
84	2.00E-01	2.00E-01	-1.55E-02	7.38E-03	4.68E-14	11.96	5.13E-14	8.91E-15	3.89E-18	1.07E-25	1.85E-01	7.38E-03	1.55E-02
85	2.00E-01	2.00E-01	-2.04E-02	7.38E-03	4.68E-14	12.08	3.89E-14	5.13E-15	1.70E-18	3.55E-26	1.85E-01	7.38E-03	2.04E-02
86	2.00E-01	2.00E-01	-2.69E-02	7.38E-03	4.68E-14	12.2	2.95E-14	2.95E-15	7.41E-19	1.18E-26	1.85E-01	7.38E-03	2.69E-02
87	2.00E-01	2.00E-01	-3.55E-02	7.38E-03	4.68E-14	12.32	2.24E-14	1.70E-15	3.24E-19	3.89E-27	1.85E-01	7.38E-03	3.55E-02
88	2.00E-01	2.00E-01	-4.68E-02	7.38E-03	4.68E-14	12.44	1.70E-14	9.77E-16	1.41E-19	1.29E-27	1.85E-01	7.38E-03	4.68E-02
89	2.00E-01	2.00E-01	-6.17E-02	7.38E-03	4.68E-14	12.56	1.29E-14	5.62E-16	6.17E-20	4.27E-28	1.85E-01	7.38E-03	6.17E-02
90	2.00E-01	2.00E-01	-8.13E-02	7.38E-03	4.68E-14	12.68	9.77E-15	3.24E-16	2.69E-20	1.41E-28	1.85E-01	7.38E-03	8.13E-02
91	2.00E-01	2.00E-01	-1.07E-01	7.38E-03	4.68E-14	12.8	7.41E-15	1.86E-16	1.18E-20	4.68E-29	1.85E-01	7.38E-03	1.07E-01
92	2.00E-01	2.00E-01	-1.41E-01	7.38E-03	4.68E-14	12.92	5.62E-15	1.07E-16	5.13E-21	1.55E-29	1.85E-01	7.38E-03	1.41E-01
93	2.00E-01	2.00E-01	-1.86E-01	7.38E-03	4.68E-14	13.04	4.27E-15	6.17E-17	2.24E-21	5.13E-30	1.85E-01	7.38E-03	1.86E-01
94	2.00E-01	2.00E-01	-2.46E-01	7.38E-03	4.68E-14	13.16	3.24E-15	3.55E-17	9.77E-22	1.70E-30	1.85E-01	7.38E-03	2.46E-01
95	2.00E-01	2.00E-01	-3.24E-01	7.38E-03	4.68E-14	13.28	2.46E-15	2.04E-17	4.27E-22	5.62E-31	1.85E-01	7.38E-03	3.24E-01
96	2.00E-01	2.00E-01	-4.27E-01	7.38E-03	4.68E-14	13.4	1.86E-15	1.18E-17	1.86E-22	1.86E-31	1.85E-01	7.38E-03	4.27E-01
97	2.00E-01	2.00E-01	-5.62E-01	7.38E-03	4.68E-14	13.52	1.41E-15	6.76E-18	8.13E-23	6.17E-32	1.85E-01	7.38E-03	5.62E-01
98	2.00E-01	2.00E-01	-7.41E-01	7.38E-03	4.68E-14	13.64	1.07E-15	3.89E-18	3.55E-23	2.04E-32	1.85E-01	7.38E-03	7.41E-01

Table S4. HySS2009 - Concentration table. (continued)

99	2.00E-01	2.00E-01	-9.77E-01	7.38E-03	4.68E-14	13.76	8.13E-16	2.24E-18	1.55E-23	6.76E-33	1.85E-01	7.38E-03	9.77E-01
100	2.00E-01	2.00E-01	-1.29E+00	7.38E-03	4.68E-14	13.88	6.17E-16	1.29E-18	6.76E-24	2.24E-33	1.85E-01	7.38E-03	1.29E+00
101	2.00E-01	2.00E-01	-1.70E+00	7.38E-03	4.68E-14	14	4.68E-16	7.41E-19	2.95E-24	7.41E-34	1.85E-01	7.38E-03	1.70E+00

References

1. Venkateswarlu, C.; Das, M.S.; Athavale, V.T. Studies of gallic acid complexes with metals and their analytical applications. Part I. Spectrophotometric Investigation. *Proceedings of the Indian Academy of Sciences - Section A* **1954**, *40*, 260-269.
2. Varde, M.S.; Athavale, V.T. Studies of gallic acid complexes with metals and their analytical applications – Part II. A Spectrophotometric study of molybdenum complexes. *Proceedings of the Indian Academy of Sciences - Section A* **1956**, *44*, 228-240.
3. Venkateswarlu, C.; Das, M.S.; Athavale, V.T. Studies of gallic acid complexes with metals and their analytical applications - Part III. Spectrophotometric estimation of gallic acid. *Proceedings of the Indian Academy of Sciences - Section A* **1956**, *44*, 241-246.
4. Elinany, G.A.; Ebeid, F.M.; Zahra, A.M.; Ziedan, F.I. POLAROGRAPHY OF METAL-GALLIC COMPLEXES. *Journal of Electroanalytical Chemistry* **1976**, *72*, 363-369.
5. Powell, H.K.J.; Taylor, M.C. Interactions of Iron(II) and Iron(III) with Gallic Acid and its Homologues: a Potentiometric and Spectrophotometric Study. *Australian Journal of Chemistry* **1982**, *35*, 739-756.
6. Sandmann, B.J.; Chien, M.H.; Sandmann, R.A. Stability constant of calcium, magnesium and zinc gallate using a divalent ion-selective electrode. *Analytical Letters* **1985**, *18*, 149-159.
7. Fazary, A.E.; Taha, M.; Ju, Y.-H. Iron Complexation Studies of Gallic Acid. *Journal of Chemical & Engineering Data* **2008**, *54*, 35-42.
8. Zaccaron, S.; Ganzerla, R.; Bortoluzzi, M. Iron complexes with gallic acid: a computational study on coordination compounds of interest for the preservation of cultural heritage. *Journal of Coordination Chemistry* **2012**, *66*, 1709-1719.
9. Masoud, M.S.; Ali, A.E.; Haggag, S.S.; Nasr, N.M. Spectroscopic studies on gallic acid and its azo derivatives and their iron(III) complexes. *Spectrochimica Acta Part A: Molecular and Biomolecular Spectroscopy* **2014**, *120*, 505-511.
10. Gao, F.; Zheng, D.; Tanaka, H.; Zhan, F.; Yuan, X.; Gao, F.; Wang, Q. An electrochemical sensor for gallic acid based on Fe_2O_3/electro-reduced graphene oxide composite: Estimation for the antioxidant capacity index of wines. *Materials Science and Engineering C-Materials for Biological Application* **2015**, *57*, 279-287.
11. Daduang, J.; Palasap, A.; Daduang, S.; Boonsiri, P.; Suwannalert, P.; Limpaiboon, T. Gallic acid enhancement of gold nanoparticle anticancer activity in cervical cancer cells. *Asian Pacific Journal of Cancer Prevention* **2015**, *16*, 169-174.
12. Rahim, S.A.; Hussain, S.; Farooqui, M. Protonation Equilibria of Gallic Acid (GA) and Stability Constants of Its Complexes with Transition Metal Ions in Aqueous Media. *Journal of Chemical, Biological and Physical Sciences* **2017**, *7*, 267-273.
13. Wang, Y.; Zhang, J.; Zhang, C.; Li, B.; Wang, J.; Zhang, X.; Li, D.; Sun, S.-K. Functional-Protein-Assisted Fabrication of Fe–Gallic Acid Coordination Polymer Nanonetworks for Localized Photothermal Therapy. *ACS Sustainable Chemistry & Engineering* **2018**, *7*, 994-1005.
14. Khan, A.N.; Hassan, M.N.; Khan, R.H. Gallic acid: A naturally occurring bifunctional inhibitor of amyloid and metal induced aggregation with possible implication in metal-based therapy. *Journal of Molecular Liquids* **2019**, *285*, 27-37.
15. Jing, Z.; Li, M.; Wang, H.; Yang, Z.; Zhou, S.; Ma, J.; Meng, E.; Zhang, H.; Liang, W.; Hu, W.; Wang, X.; Fu, X. Gallic acid-gold nanoparticles enhance radiation-induced cell death of human glioma U251 cells. *IUBMB Life* **2021**, *73*, 398-407.
16. Frešer, F.; Hostnik, G.; Tošović, J.; Bren, U. Dependence of the Fe(II)-Gallic Acid Coordination Compound Formation Constant on the pH. *Foods* **2021**, *10*, 2689.

17. El-Megharbel, S.M.; Hamza, R.Z. Synthesis, spectroscopic characterizations, conductometric titration and investigation of potent antioxidant activities of gallic acid complexes with Ca (II), Cu (II), Zn(III), Cr(III) and Se (IV) metal ions. *Journal of Molecular Liquids* **2022**, *358*, 119196.
18. Novaković, T.B.; Pavlović, S.M.; Pagnacco, M.C.; Banković, P.T.; Mojović, Z.D. The Application of Alumina for Electroanalytical Determination of Gallic Acid. *Electrocatalysis* **2023**, *14*, 18-28.
19. Moralles, V.A.; Davolos, M.R.; Cebim, M.A. Europium(III) and gallic acid ligand coordination compounds: synthesis, characterization, photophysical processes, and optimization of luminescent properties. *Inorganica Chimica Acta* **2023**, *558*, 121748.
20. Şişmanoğlu, T.; İçhedef, Ç.; Akdut, G.; Soylu, G.S.P.; Medine, E.İ.; Teksöz, S. Complexation of gallic acid involving La^{3+}, Sm^{3+}, Th^{4+} and UO_2^{2+} ions in aqueous solution by potentiometry at various temperatures. *Journal of Radioanalytical and Nuclear Chemistry* **2024**, DOI: https://doi.org/10.1007/s10967-024-09829-4
21. Goman, D.; Stanković, A.; Galović, O.; Džakula, B.N.; Kontrec, J.; Medvidović-Kosanović, M. Complexation of gallic acid with calcium: electrochemical, potentiometric, and UV-VIS studies. *Analytical Methods* **2024**, *16*, 391-395.
22. Hou, X.; Zhang, L.; Chen, Y.; Liu, Z.; Zhao, X.; Lu, B.; Luo, Y.; Qu, X.; Musskaya, O.; Glazov, I.; Kulak, A.I.; Chen, F.; Zhao, J.; Zhou, Z.; Zheng, L. Photothermal switch by gallic acid-calcium grafts synthesized by coordination chemistry for sequential treatment of bone tumor and regeneration. *Biomaterials* **2025**, *312*, 122724.
23. Cherepanov, P.V.; Rahim, M.A.; Bertleff-Zieschang, N.; Sayeed, M.A.; O'Mullane, A.P.; Moulton, S.E.; Caruso, F. Electrochemical Behavior and Redox-Dependent Disassembly of Gallic Acid/Fe III Metal–Phenolic Networks. *ACS Applied Materials & Interfaces* **2018**, *10*, 5828-5834.
24. Santoso, S.P.; Bundjaja, V.; Angkawijaya, A.E.; Gunarto, C.; Go, A.W.; Yuliana, M.; Tran-Nguyen, P.L.; Hsieh, C.-W.; Ju, Y.-H. One-step synthesis of nitrogen-grafted copper-gallic acid for enhanced methylene blue removal. *Scientific Reports* **2021**, *11*, 12021.
25. Wasuwanich, P.; Fan, G.; Burke, B.; Furst, A.L. Metal-phenolic networks as tuneable spore coat mimetics. *Journal of Materials Chemistry B* **2022**, *10*, 7600-7606.
26. Sharma, S.; Mittal, D.; Verma, A.K.; Roy, I. Copper-Gallic Acid Nanoscale Metal–Organic Framework for Combined Drug Delivery and Photodynamic Therapy. *ACS Applied Bio Materials* **2019**, *2*, 2092–2101.
27. Wu, J.; Ouyang, D.; He, Y.; Su, H.; Yang, B.; Li, J.; Sun, Q.; Lin, Z.; Cai, Z. Synergistic Effect of Metal–Organic Framework/Gallic Acid in Enhanced Laser Desorption/Ionization Mass Spectrometry. *ACS Applied Materials & Interfaces* **2019**, *11*, 38255–38264.
28. Azhar, B.; Angkawijaya, A.E.; Santoso, S.P.; Gunarto, C.; Ayucitra, A.; Go, A.W.; Tran-Nguyen, P.L.; Ismadji, S.; Ju, Y.-H. Aqueous synthesis of highly adsorptive copper–gallic acid metal–organic framework. *Scientific Reports* **2020**, *10*, 19212.
29. El-Shahawy, A.A.G.; Dief, E.M.; El-Dek, S.I.; Farghali, A.A.; El-Ela, F.I.A. Nickel-gallate metal–organic framework as an efficient antimicrobial and anticancer agent: in vitro study. *Cancer Nanotechnology* **2023**, *14*, 60.
30. Azhar, B.; Avian, C.; Tiwikrama, A.H. Green synthesis optimization with artificial intelligence studies of copper–gallic acid metal–organic framework and its application in dye removal from wastewater. *Journal of Molecular Liquids* **2023**, *389*, 122844.
31. Hamedi, H.; Javanbakht, S.; Mohammadi, R. In-situ synthesis of copper-gallic acid metal–organic framework into the gentamicin-loaded chitosan hydrogel bead: A synergistic enhancement of antibacterial properties. *Journal of Industrial and Engineering Chemistry* **2024**, *133*, 454-463.
32. Gu, L.; Li, X.; Chen, G.; Yang, H.; Qian, H.; Pan, J.; Miao, Y.; Li, Y. A glutathione-activated bismuth-gallic acid metal-organic framework nano-prodrug for enhanced sonodynamic therapy of breast tumor. *Journal of Colloid and Interface Science* **2025**, *679*, 214-223.
